# TCGA-Informed Spatial Profiling Reveals Peripheral CD147 Expression at the Invasive Tumor Front as a Prognostic Indicator in OSCC

**DOI:** 10.3390/ijms27052172

**Published:** 2026-02-25

**Authors:** Felix Nieberle, Steffen Spoerl, Quirin Strotzer, Robin Hartmann, Ramona Erber, Silvia Spoerl, Johannes G. Schuderer, Katja Himmelstoß, Johannes Meier, Tobias Ettl, Torsten E. Reichert, Juergen Taxis

**Affiliations:** 1 Department of Cranio-Maxillofacial Surgery, University Hospital Regensburg, 93053 Regensburg, Germany; felix.nieberle@klinik.uni-regensburg.de (F.N.); steffen.spoerl@klinik.uni-regensburg.de (S.S.); johannes.schuderer@klinik.uni-regensburg.de (J.G.S.); katja.himmelstoss@klinik.uni-regensburg.de (K.H.); johannes.meier@klinik.uni-regensburg.de (J.M.); tobias.ettl@klinik.uni-regensburg.de (T.E.); torsten.reichert@klinik.uni-regensburg.de (T.E.R.); 2 Department of Radiology, University Hospital Regensburg, 93053 Regensburg, Germany; quirin.strotzer@klinik.uni-regensburg.de; 3 Department of Oral and Maxillofacial Surgery and Facial Plastic Surgery, Ludwig-Maximilians-University (LMU), Lindwurmstrasse 2a, 80337 Munich, Germany; robin.hartmann@med.uni-muenchen.de; 4 Institute of Pathology, University Regensburg, 93053 Regensburg, Germany; ramona.erber@klinik.uni-regensburg.de; 5 Institute of Pathology, University Hospital Erlangen, Friedrich-Alexander-University Erlangen-Nürnberg (FAU), 91054 Erlangen, Germany; 6 Department of Internal Medicine 5—Haematology and Oncology, University Hospital Erlangen, Friedrich-Alexander-University Erlangen-Nürnberg (FAU), 91054 Erlangen, Germany; silvia.spoerl@uk-erlangen.de

**Keywords:** oral squamous cell carcinoma, CD147, Basigin, prognosis, tumor microenvironment, immune evasion, biomarker, tissue microarray, therapy resistance, TCGA

## Abstract

Oral squamous cell carcinoma (OSCC) remains a major cause of cancer-related mortality worldwide, with limited biomarker-driven tools for risk stratification. CD147 is a membrane glycoprotein implicated in tumor metabolism, invasion, immune evasion, and therapy resistance. This study aimed to evaluate the prognostic and predictive relevance of CD147 expression in distinct tumor compartments of OSCC. Formalin-fixed tumor samples from 229 OSCC patients were analyzed via tissue microarray and immunohistochemistry to assess CD147 expression in the tumor center, periphery, and adjacent mucosa. Associations with clinicopathological parameters, survival, and therapy response were evaluated using non-parametric statistical tests, Kaplan–Meier, multivariate Cox, and binary logistic regression analyses. Complementary transcriptomic and immunological analyses were performed using The Cancer Genome Atlas (TCGA), the University of Alabama at Birmingham Cancer data analysis (UALCAN), Tumor and Immune System Interaction Database (TISIDB), and the Genotype-Tissue Expression (GTEx) project’s datasets. Low CD147 expression in the tumor invasive front was independently associated with improved overall survival, while expression in the tumor center or mucosa showed no prognostic value. No significant associations between CD147 and adjuvant therapy response were identified. TCGA-based analyses confirmed CD147 overexpression in tumors and its correlation with immunosuppressive signaling and resistance-associated transcriptional networks. Peripheral CD147 expression serves as a compartment-specific, independent prognostic marker in OSCC in this retrospective single-center cohort. Its spatially restricted prognostic relevance and association with immune modulation and therapy resistance highlight CD147 as a promising candidate for future biomarker-driven and therapeutic strategies.

## 1. Introduction

Despite the immeasurable efforts and investments in cancer research, cancer is estimated to become the overall leading cause of death in the 21st century worldwide [1]. Herein, head and neck cancer, with its predominant form of oral squamous cell carcinoma (OSCC), is projected to be among the top ten types of cancer [1,2,3]. Even after the formulation of the ‘hallmarks of cancer’ by Hanahan and Weinberg more than two decades ago, their subsequent further refinement and thereby specification of therapeutic targets, only an average survival rate of approximately 50% has been achieved in patients suffering from OSCC [1,4].

Current therapeutic options are diverse and primarily include surgical resection, radiotherapy, chemotherapy, oncogene-targeted therapy, immunotherapy, or combinations thereof, depending on tumor stage [5]. Given the stage-dependent and multimodal nature of OSCC care, selecting an appropriate therapeutic strategy and coordinating treatment across specialties is challenging, making close interdisciplinary collaboration indispensable. Accordingly, multidisciplinary team (MDT) involvement and structured care pathways, including integrating pathology-driven risk stratification, adjuvant (chemo)radiotherapy, rehabilitation, dental and nutritional support, and speech/swallowing therapy, are increasingly emphasized to optimize outcomes. Systematic evidence across oncology suggests that MDT meetings can improve diagnostic accuracy, staging completeness, and adherence to guideline-concordant multimodality treatment, although survival effects vary by setting and study design [6].

OSCC commonly emerges in the context of established exogenous risk factors, including tobacco exposure, alcohol use, and region-specific carcinogens such as areca nut/betel quid. Additionally, viral cofactors such as HPV infections are etiologically relevant in certain settings [7,8]. However, OSCC also occurs in patients without these exposures, suggesting that endogenous, tumor-intrinsic factors and microenvironmental programs, among others, contribute substantially to disease development, a concept highlighted in the recent literature [5,9,10].

In the context of endogenous factors, cluster of differentiation 147 (CD147) has been investigated in numerous tumor entities, as well as in OSCC, since CD147 overexpression is associated with several hallmarks of cancer, including sustaining proliferation, resisting cell death, enabling invasion and metastasis, inducing angiogenesis, and immune evasion, among others [11,12].

CD147, also known as extracellular matrix metalloproteinase inducer (EMMPRIN) or Basigin (BSG), is a type I transmembrane glycoprotein belonging to the immunoglobulin superfamily. It has been shown to contribute to proliferation by interacting with glucose transporter 1 (GLUT1), monocarboxylate transporter 1 (MCT1), MCT3, and MCT4. GLUT1 facilitates sufficient glucose uptake into tumor cells, thereby sustaining glycolytic energy production. At the same time, MCT1, MCT3, and MCT4 mediate lactate export to regulate pH balance and thereby support the metabolic reprogramming characteristic of the Warburg effect [11,13,14].

Beyond the metabolic functions, invasiveness and metastasis can be promoted by creating local conditions that enable matrix degradation and tissue remodeling. A key mechanism involves the CD147-mediated induction and vesicular secretion of matrix metalloproteinases (MMPs), such as MMP-2, -3, and -9, by tumor cells. These MMPs interact with receptors on fibroblasts and other stromal cells in the environment to amplify proteolytic activity, resulting in the breakdown of the extracellular matrix, triggering the epithelial–mesenchymal transition (EMT) and enhancing vascular permeability, thereby fostering tumor cell invasion and metastatic dissemination [11].

CD147 also contributes to the induction of angiogenesis by promoting the release of matrix-bound angiogenic factors via MMPs and by enhancing the production of soluble vascular endothelial growth factor (VEGF) isoforms together with their receptor VEGFR-2, for which CD147 has also been reported to function as a co-receptor. Moreover, elevated extracellular lactate resulting from glycolytic reprogramming has been shown to stimulate angiogenesis further [13,15].

Beyond these established oncogenic functions, CD147 expression has also been closely linked to the development of chemoresistance [12,16,17,18]. In OSCC cell line models, enforced CD147 upregulation is correlated with reduced chemosensitivity and increased resistance to apoptosis. One mechanistic explanation involves receptor crosstalk between CD147 and CD44, a hyaluronic acid receptor and marker of cancer stem cells [18]. The CD147-CD44 axis promotes the assembly of plasma membrane complexes that regulate downstream signaling pathways associated with poor survival and resistance to cytotoxic agents [16,18,19,20].

In addition, hypoxia-inducible factor 1-alpha (HIF-1α) has emerged as a critical regulator of chemoresistance, as well as tumor growth and angiogenesis [21,22,23]. Evidence suggests a regulatory interplay among CD147, HIF-1α, and cyclophilin A (CypA). In lung cancer, HIF-1α upregulates CypA, conferring protection against cellular stress and chemotherapy [23]. Moreover, CypA interacts with CD147 to promote cancer cell growth and poor prognosis, as shown in pancreatic carcinoma [20]. Through this feed-forward loop, HIF-1α may regulate CD147 signaling via CypA, thereby linking hypoxia to enhanced tumor progression and therapy resistance.

Therefore, based on these findings, we focused our investigations on the role of CD147 in OSCC, with particular emphasis on its contribution to prognosis and resistance to adjuvant therapy.

## 2. Results

### 2.1. Clinicopathological Characteristics and CD147 Expression Patterns

A total of 229 cases of OSCC were included in this study, with baseline clinicopathological characteristics being summarized in Table 1.

The majority of patients were male (71.2%) and younger than 70 years at diagnosis (78.2%). A history of tobacco use was reported in 78.2% of patients, and alcohol consumption in 70.7%. The most frequent tumor localization was the floor of the mouth (47.2%), followed by the lower alveolar ridge and gingiva (21.4%).

Regarding tumor staging, 65.9% of patients presented with T1/T2 tumors, while 34.1% were T3/T4 carcinomas. Lymph node involvement (N+) was observed in 44.5% of patients, and 60.3% were diagnosed with UICC stage III/IV disease. Histologically, most tumors were classified as G2 (83.0%). Vascular invasion (V1) and perineural invasion (Pn1) were relatively rare, occurring in 4.4% and 3.1% of cases, respectively. Recurrence occurred in 25.3% of patients, and 65.5% had died by the end of the follow-up period of 15.9 years. Adjuvant treatment was administered in 52.4% of patients, with 37.6% receiving radiotherapy and 14.8% receiving combined radiochemotherapy.

**Figure 1:**
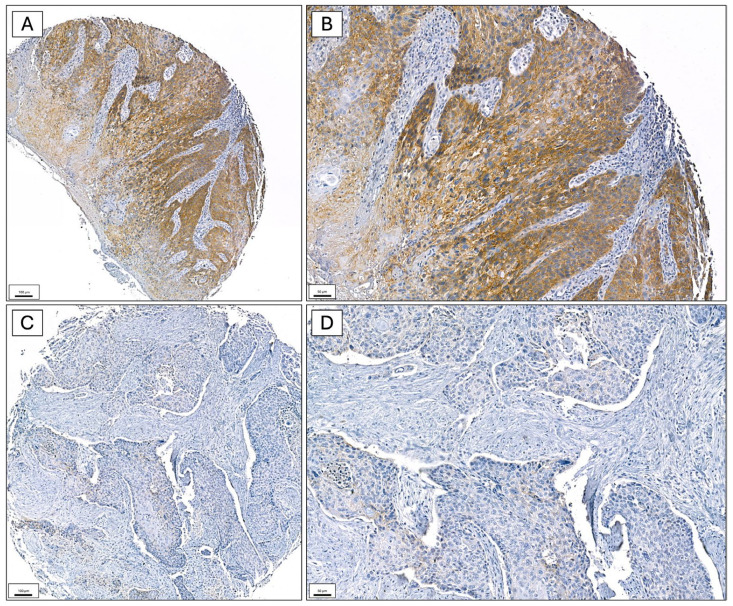
Representative immunohistochemical staining of CD147 at the tumor invasion front of oral squamous cell carcinoma. (**A**,**B**) Case with high CD147 expression (immunoscore 285) showing strong membranous staining of neoplastic squamous cells at low ((**A**), ×100) and intermediate ((**B**), ×200) magnification. (**C**,**D**) Case with low CD147 expression (immunoscore 20) demonstrating weak to absent membranous staining at low ((**C**), ×100) and intermediate ((**D**), ×200) magnification. CD147 immunoreactivity was visualized with DAB (brown) and nuclei were counterstained with hematoxylin (blue).

**Figure A1:**
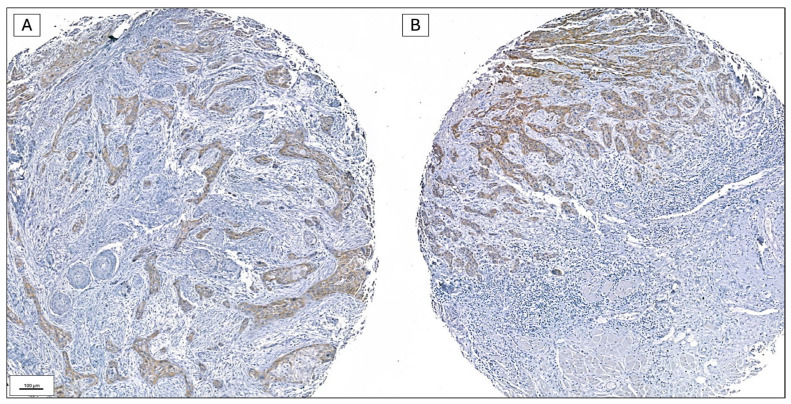
Representative immunohistochemical staining of CD147 in oral squamous cell carcinoma comparing the tumor center and the tumor invasion front. (**A**) Tumor center and (**B**) tumor invasion front of the same case, both showing similar CD147 expression. Original magnification ×100. CD147 immunoreactivity was visualized with DAB (brown) and nuclei were counterstained with hematoxylin (blue).

CD147 expression was assessed semiquantitatively using an immunoscore in three distinct tissue compartments, as mentioned below. In the tumor center, CD147 expression was negative in 14.4% of cases, low in 22.7%, and high in 51.1%. In the tumor periphery, negative expression was observed in 14.8% of cases, low in 16.2%, and high in 48.9% (representative staining of CD147 expression in the tumor invasion front shown in Figure 1, comparative expression of CD147 in tumor center and tumor periphery shown in Figure A1). In contrast, the adjacent mucosa showed predominantly low expression levels, with 23.6% of cases being negative, 31.0% having low, and 0.4% having high immunoscore. Due to insufficient tissue quality or missing tissue in the respective section, immunostaining could not be reliably evaluated in 27 cases in the tumor center, 46 cases in the tumor periphery, and 103 cases in the adjacent mucosa. These cases were excluded from the corresponding immunoscore analyses. Overall, high CD147 expression was most frequently detected in tumor compartments, whereas expression in non-tumorous mucosa was largely absent or low. This pattern suggests a tumor-specific upregulation of CD147.

### 2.2. Correlation and Trend Analysis of CD147 Immunoscore with Clinicopathologic Features

The association between CD147 immunoscore and clinicopathological parameters was evaluated separately for the tumor center, tumor periphery, and adjacent mucosa, as shown in Table 2, Table 3 and Table 4.

In the tumor center (*n* = 202), CD147 immunoscore showed no statistically significant association with any clinicopathological feature. While vascular invasion displayed a near-significant difference (Mann–Whitney U test, *p* = 0.062), no correlation was found with UICC stage, nodal status, grading, or recurrence (Table 2).

CD147 immunoscore in the tumor periphery (*n* = 183) similarly did not correlate significantly with clinicopathological parameters (Table 3).

In the adjacent mucosa (*n* = 126), a significant association was observed between CD147 immunoscore and age at diagnosis (Mann–Whitney U test, *p* = 0.026), with older patients showing higher CD147 expression. No significant differences were detected for gender, T-status, N-status, UICC stage, grading, or lymphovascular, vascular, or perineural invasion. Likewise, the adjuvant treatment modality did not affect mucosal CD147 immunoscore (Kruskal–Wallis test, *p* = 0.960) (Table 4).

To explore potential monotonic trends between tumor stage and CD147 expression, Jonckheere-Terpstra tests were performed for both UICC and T classifications. No significant monotonic trend in CD147 immunoscore was observed across UICC stages for any of the analyzed tissue compartments (tumor center: *z* = 0.189, *p* = 0.850; tumor periphery: *z* = 0.586, *p* = 0.558; mucosa: *z* = 1.160, *p* = 0.246). Similarly, no statistically significant trend was detected across T-stages. However, a weak tendency toward increasing CD147 expression with higher T-category was observed in the tumor center (*z* = 1.854, *p* = 0.064) and tumor periphery (*z* = 1.454, *p* = 0.146). These findings suggest that while CD147 expression may exhibit a mild increase with advancing local tumor extent, the overall relationship between CD147 immunoscore and tumor stage did not reach statistical significance.

Collectively, these findings indicate that CD147 expression, as quantified by immunoscore, is mainly independent of standard clinicopathological parameters, except for a potential trend toward higher expression with increasing tumor stage and patient age.

### 2.3. Kaplan–Meier Survival Analysis According to CD147 Immunoscore

**Figure 2:**
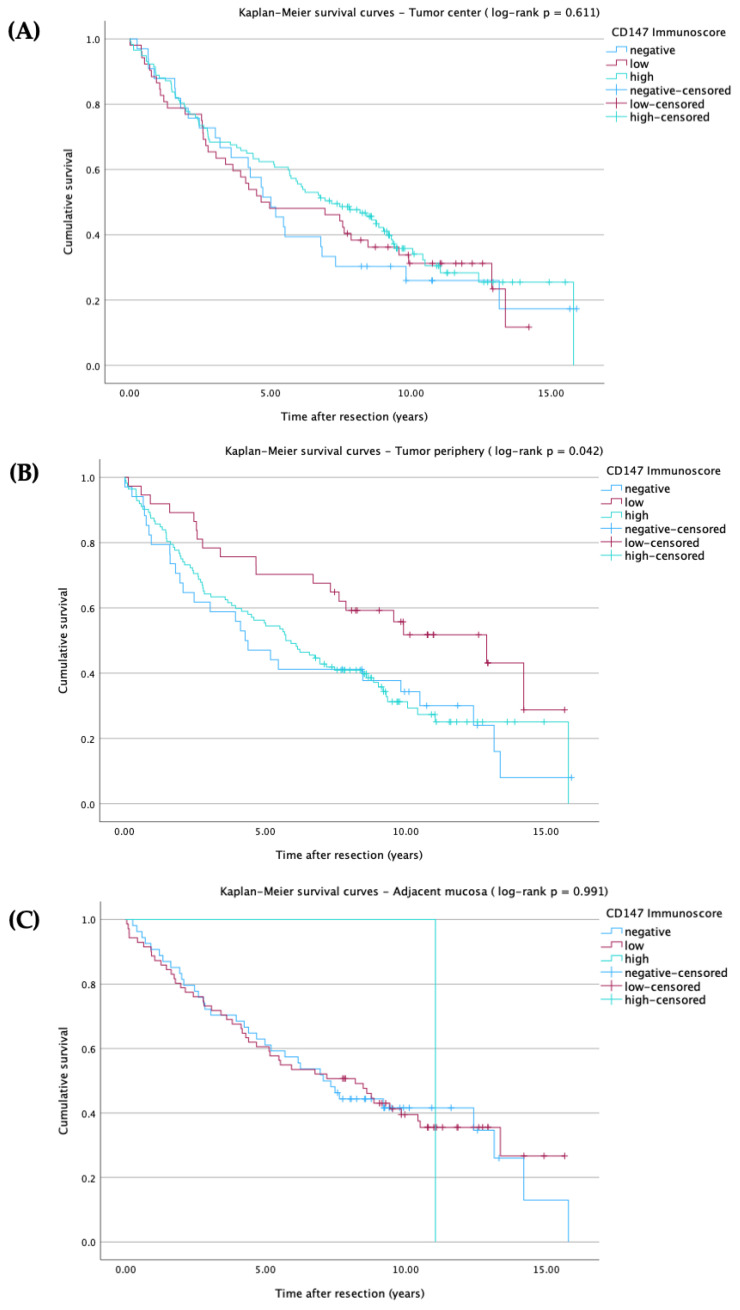
Kaplan–Meier survival analysis according to CD147 immunoscore levels, cross marks (+) indicate censored observations. Survival probabilities were estimated using the Kaplan–Meier method, and comparisons between CD147 immunoscore groups (negative, low, high) were performed using the Mantel–Cox log-rank test. Hazard ratios (HR) taken from univariate cox regression analyses. (**A**) Overall survival according to CD147 immunoscore in the tumor center (log-rank *p* = 0.611, HR = 0.923, 95% CI: 0.554–1.537). (**B**) Overall survival according to CD147 immunoscore in the tumor periphery (log-rank *p* = 0.042, HR = 0.514, 95% CI: 0.284–0.930). (**C**) Overall survival according to CD147 immunoscore in the adjacent mucosa (log-rank *p* = 0.991, HR = 0.971, 95% CI: 0.621–1.518).

Kaplan–Meier survival analysis was performed to assess overall survival according to CD147 immunoscore (negative, low, high) in the tumor center, tumor periphery, and adjacent mucosa (Figure 2A–C).

These analyses revealed no significant differences in survival for CD147 expression in the tumor center (log-rank *p* = 0.611; HR = 0.923, 95% CI: 0.554–1.537; Figure 2A) or in the adjacent mucosa (log-rank *p* = 0.991; HR = 0.971, 95% CI: 0.621–1.518; Figure 2C). In contrast, the CD147 immunoscore at the tumor periphery was significantly associated with overall survival (log-rank *p* = 0.042, HR = 0.514, 95% CI: 0.284–0.930; Figure 2B), with patients exhibiting low peripheral CD147 expression showing a survival advantage compared with the other immunoscore groups.

**Figure 3:**
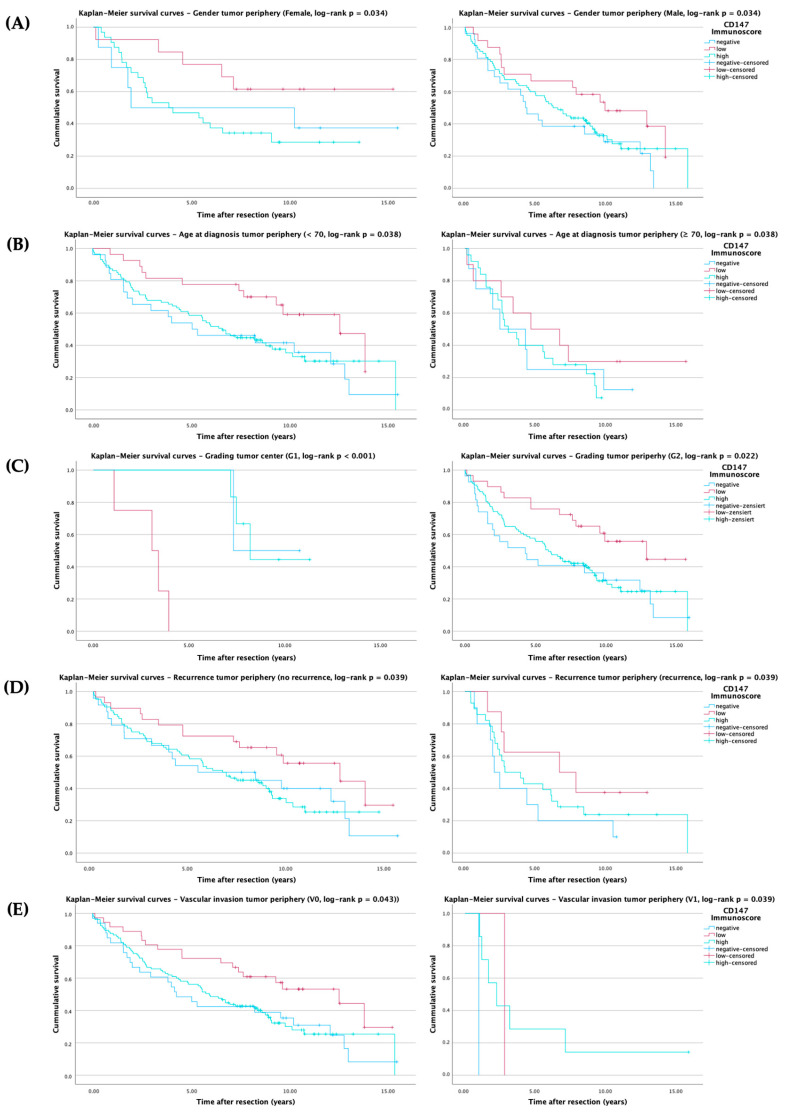
Kaplan–Meier survival analysis according to CD147 immunoscore (blue = negative, red = low, cyan = high), cross marks (+) indicate censored observations. Survival probabilities were estimated using the Kaplan–Meier method, and comparisons between CD147 immunoscore groups were performed using the log-rank test. (**A**) Subgroup Gender survival analysis according to CD147 immunoscore in the tumor periphery (log-rank *p* = 0.034). (**B**) Subgroup Age at diagnosis survival analysis according to CD147 immunoscore in the tumor periphery (log-rank *p* = 0.038). (**C**) Subgroup Grading survival analysis according to CD147 immunoscore in the tumor center and periphery (log-rank *p* < 0.001 and *p* = 0.022, respectively). (**D**) Subgroup Recurrence survival analysis according to CD147 immunoscore in the tumor periphery (log-rank *p* = 0.039). (**E**) Subgroup Vascular invasion survival analysis according to CD147 immunoscore in the tumor periphery (log-rank *p* = 0.043).

To further explore these associations, subgroup analyses were performed and stratified by clinicopathological parameters and tissue compartments. Statistically significant overall survival analyses are listed in Table A1 and illustrated in Figure 3A–E, while other parameters showed no significant associations and are not presented. Given the number of stratified comparisons across clinicopathological subgroups and tissue compartments, and the reduced sample size in several strata, these subgroup Kaplan–Meier analyses should be considered exploratory and hypothesis-generating. Therefore, *p*-values are reported descriptively and were not adjusted for multiple testing. Findings should thus be interpreted with caution, particularly when subgroup sizes are small.

Herein, Kaplan–Meier analyses revealed that, in the tumor periphery, overall survival differed by gender (χ^2^ = 13.992, *p* = 0.034), with female patients benefiting more from low CD147 expression than male patients.

The same results could be seen in the age-at-diagnosis subgroups, as patients > 70 years of age (χ^2^ = 6.540, *p* = 0.038), compared with patients aged ≤70 years, showed poorer outcomes linked to higher peripheral CD147 expression.

Regarding tumor grading, a significant association between CD147 immunoscore and overall survival was observed in well-differentiated (G1) tumors of the tumor center (χ^2^ = 13.992, *p* = < 0.001). In this subgroup (*n* = 12), patients with low CD147 expression demonstrated shorter overall survival compared to those with high or negative expression levels. Although this finding contrasts with the expected direction of association, the small number of cases in this subgroup likely limits the robustness of this observation. The distribution of cases (two negative, four low, and six high expression) and the complete event occurrence in the low-expression group (4/4 deaths, zero censored) suggest that the apparent survival disadvantage of low CD147 expression may primarily reflect sample size constraints rather than a biological effect. In moderately differentiated (G2) and poorly differentiated (G3) tumors of the tumor center, no significant associations were detected (*p* = 0.729 and *p* = 0.752, respectively).

In the tumor periphery, survival also differed significantly among CD147 immunoscore groups in moderately differentiated tumors (*χ*^2^ = 7.629, *p* = 0.022), where low CD147 expression again correlated with improved overall survival.

However, no significant survival differences were detected for G1 (*p* = 0.938) or G3 (*p* = 0.918) tumors in the tumor periphery.

Additionally, recurrence (*χ*^2^ = 6.478, *p* = 0.039) and vascular invasion (*χ*^2^ = 6.299, *p* = 0.043) were significantly linked to survival differences, with low CD147 expression being associated with longer survival times.

While most other parameters showed no significant associations, several subgroups in the tumor periphery demonstrated near-significant trends, suggesting a biological and therefore prognostic relevance of CD147 expression.

Among patients without lymph node metastasis (N0), there was a trend toward improved survival in those cases with low peripheral CD147 immunoscore compared with high or negative expression (*p* = 0.090). A similar trend was observed in OSCC patients without lymph vessel invasion (L0) at the tumor periphery (*p* = 0.071), in which low CD147 immunoscore was associated with prolonged survival.

In UICC stage IV tumors, a comparable tendency was noted (*p* = 0.104), with patients showing low CD147 expression exhibiting prolonged overall survival.

Although these trends did not reach formal statistical significance, they consistently pointed toward a potential survival benefit associated with reduced CD147 expression in less invasive and advanced-stage tumor settings.

Collectively, the data support the prognostic utility of peripheral CD147 expression in OSCC, indicating its potential role in patient risk stratification. Low CD147 expression emerges as a marker of favorable prognosis across multiple clinicopathological subgroups.

### 2.4. Univariate and Multivariate Regression Analysis of Prognostic Factors for Survival

To identify prognostic factors for patient survival, Cox proportional hazard analyses were conducted, incorporating clinicopathological parameters and CD147 expression levels. Univariate and multivariate Cox proportional-hazards results are shown in Table 5 and were used to examine time-to-event outcomes, focusing on overall survival. Multivariate Cox models were adjusted for relevant clinical covariates, including CD147 immunoscore levels of the tumor periphery, age, T-status, N-status, UICC stage, recurrence, lymph vessel invasion, vessel invasion, and adjuvant therapy.

In univariate Cox proportional-hazards analysis, several parameters were significantly associated with reduced survival, including older age at diagnosis (≥70 years; *p* = 0.002, hazard ratio (HR) = 1.778, 95% confidence interval (CI): 1.235–2.560), T-status (T4; *p* = 0.001, HR = 2.028, 95% CI: 1.324–3.106), positive nodal status (N+; *p* = 0.004, HR = 1.601, 95% CI: 1.161–2.207), UICC stage III (*p* = 0.048, HR = 1.735, 95% CI: 1.004–3.000), UICC stage IV (*p* = 0.007, HR = 1.822, 95% CI: 1.175–2.826), tumor recurrence (*p* = 0.004, HR = 1.675, 95% CI: 1.181–2.375), lymph vessel invasion (L1; *p* = < 0.001, HR = 1.958, 95% CI: 1.321–2.901), vessel invasion (V1; *p* = 0.020, HR = 2.234, 95% CI: 1.135–4.399), and adjuvant radiotherapy (*p* = 0.018, HR = 1.520, 95% CI: 1.074–2.152). Low CD147 expression in the tumor periphery showed a significant positive correlation with survival (low; *p* = 0.028, HR = 0.514, 95% CI: 0.284–0.930), whereas expression in the tumor center or the surrounding mucosa did not show any significant associations.

In multivariate Cox analysis, the prognostic impact of low CD147 expression in the tumor periphery remained significant (*p* = 0.024, HR = 0.492, 95% CI: 0.266–0.909). Age ≥ 70 (*p* = 0.001, HR = 2.036, 95% CI: 1.322–3.135) alongside positive nodal status (*p* = 0.020, HR = 2.253, 95% CI: 1.134–4.475), tumor recurrence (*p* = 0.021, HR = 1.613, 95% CI: 1.074–2.423), and lymph vessel invasion (*p* = 0.009, HR = 1.946, 95% CI: 1.179–3.213) also remained as independent factors for survival. No independent prognostic significance was observed for CD147 expression in the tumor center or adjacent mucosa.

In summary, low CD147 expression in the tumor periphery emerged as an independent prognostic marker for improved overall survival. In contrast, expression levels in the tumor center and mucosa showed no prognostic relevance. Classic clinicopathological factors such as older age, positive nodal status, recurrence, and lymphatic invasion also demonstrated independent associations with poorer prognosis, underscoring their continued relevance in survival prediction.

Additionally, logistic regression analyses were conducted to examine the association between CD147 immunoscore and treatment response across the adjuvant therapy settings, namely adjuvant therapy in general, radiation therapy alone, and radiochemotherapy. Therapy-response models were performed as exploratory analyses due to multiple comparisons (across compartments, immunoscore contrasts, and adjuvant treatment strata) and limited power in some subgroups, particularly in the radiochemotherapy stratum. Accordingly, odds ratios and *p*-values are presented descriptively and should be interpreted cautiously, as estimates may be unstable in small strata.

Analysis of adjuvant therapy in general revealed that CD147 expression levels in the tumor center were not significantly associated with treatment response (Table A2). Specifically, negative and low expression compared to high expression yielded non-significant odds ratios (negative vs. high: OR = 0.590, 95% CI: 0.117–2.976, *p* = 0.523; low vs. high: OR = 0.821, 95% CI: 0.277–2.435, *p* = 0.723). In the tumor periphery, no statistically significant associations were found either (negative vs. high: OR = 0.828, 95% CI: 0.208–3.302, *p* = 0.789; low vs. high: OR = 2.576, 95% CI: 0.838, *p* = 0.099), although a trend toward improved response in the “low” subgroup was observed, no statistical significance was reached. In the adjacent mucosal compartment, model convergence issues due to small group sizes resulted in extremely high and non-interpretable odds ratios, with low explained variance (Nagelkerke R^2^ < 0.05).

In the subgroup receiving radiotherapy only (Table A3), the results followed a similar pattern. In the tumor center, negative and low CD147 expression showed no significant associations with therapy response (negative vs. high: OR = 0.296, 95% CI: 0.034–2.595, *p* = 0.272; low vs. high: OR = 0.561, 95% CI: 0.135–2.333, *p* = 0.427). In the tumor periphery, results were likewise non-significant (negative vs. high: OR = 0.556, 95% CI: 0.106–2.901, *p* = 0.486; low vs. high: OR = 2.160, 95% CI: 0.579–8.055, *p* = 0.251). Within the mucosal region, only the contrast between negative and high expression could be estimated (OR = 1.250, 95% CI: 0.326–4.797, *p* = 0.745), while the “low vs. high” comparison could not be computed due to an insufficient number of cases in the “low” category. Again, no statistically significant associations emerged, and model fit remained poor (Nagelkerke R^2^ ≤ 0.049).

In patients who received adjuvant radiochemotherapy, CD147 expression levels were also not significantly linked to treatment outcomes. In the tumor center, comparisons between negative and low expression against the high category reference group showed non-significant results (negative vs. high: OR = 0.4.667, 95% CI: 0.223–97.497, *p* = 0.321; low vs. high: OR = 01.556, 95% CI: 0.256–9.469, *p* = 0.632). Similarly, in the tumor periphery, no significant associations were found (negative vs. high: OR = 3.000, 95% CI: 0.203–44.359, *p* = 424; low vs. high: OR = 4.000, 95% CI: 0.458–34.922, *p* = 0.210), although the odds ratios in the “low” group suggest a non-significant trend. In the mucosa, both comparisons were estimable. Still, they resulted in extremely large, non-interpretable odds ratios, indicating a lack of statistical stability, again due to small subgroup sizes, as shown in Table A4. As with the other models, no significant associations were found, and the model fit was poor (Nagelkerke R^2^ ≤ 0.094).

Taken together, CD147 expression levels in the tumor center, periphery, and adjacent mucosa were not significantly associated with therapy response across all adjuvant treatment modalities. While some non-significant trends were observed, particularly in the tumor periphery, none of the models demonstrated sufficient predictive value.

### 2.5. TCGA-Based Analysis of BSG Expression, Clinical Correlates, Survival Outcomes, and Immune Signatures in HNSCC

To gain deeper insights into the biological and clinical relevance of CD147 (BSG) in OSCC, transcriptomic and immunogenic data were explored and analyzed using a TCGA-based multi-level analysis of the TCGA-HNSCC cohort. While the TCGA-HNSCC dataset includes multiple anatomic subsites, OSCC represents a major subset. Therefore, the analyses presented here provide a meaningful approximation to support and contextualize the findings derived from the OSCC-specific patient cohort investigated in this study.

#### 2.5.1. BSG Expression in Normal vs. Tumor Tissue, Association with Clinicopathological Variables, and Prognostic Value

**Figure 4:**
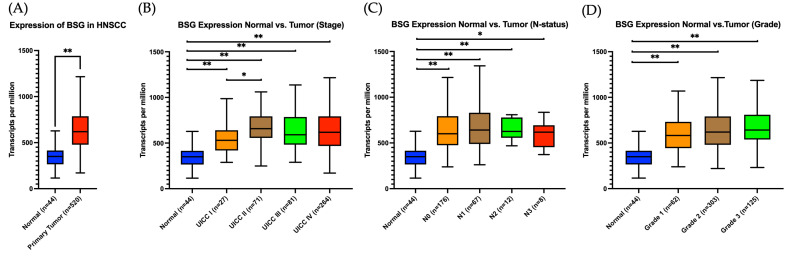
CD147 (BSG) mRNA Expression in HNSCC and Its Association with Clinicopathological Parameters (UALCAN, *n* = 520 tumors/44 normal tissues). Expression levels of BSG (CD147) were analyzed using mRNA-seq data from TCGA-HNSCC via the UALCAN web portal. (**A**) BSG expression in primary tumors (*n* = 520) was significantly elevated compared to normal tissues (*n* = 44). (**B**) Subgroup analysis by tumor Stage (UICC I-IV) revealed significantly increased expression in all stages compared to controls and a significant difference between UICC I and II. (**C**) Patients with nodal metastases (N1-N3) exhibited significantly higher BSG expression compared to controls; however, no significant difference was observed between node-negative and node-positive tumors. (**D**) Stratification by histological grade showed significantly elevated BSG expression in all tumor grades compared to healthy tissue. However, no significant differences were observed between tumor grades. Box plots represent transcript levels (transcripts per million, TPM). Statistical significance was assessed using unpaired Student’s *t*-tests. * *p* < 0.05, ** *p* < 0.001. Data accessed on 25 October 2025.

To further investigate the clinical relevance of CD147 (BSG) expression, RNA-sequencing data from the TCGA cohort in head and neck squamous cell carcinoma (HNSCC) were analyzed using the UALCAN platform and are displayed in Figure 4.

A significant upregulation of BSG transcript levels was observed in primary tumor tissues (*n* = 520) compared to normal tissues (*n* = 44) (*p* < 0.001, Figure 4A).

When stratified by UICC tumor stage, BSG expression was also significantly elevated in tumor samples compared to normal tissue (*p* < 0.001 for all stages I-IV), with the highest expression observed in UICC IV tumors (Figure 4B). A statistically significant difference was noted between stage I and stage II tumors (*p* = 0.025), but no further significant differences were observed among the higher tumor stages.

A comparison across lymph node status demonstrated significantly elevated BSG expression in all nodal-positive subgroups (N1-N3) as compared to normal tissue, with the most substantial difference observed in N1 samples (*p* < 0.001, Figure 4C). However, no statistically significant differences were detected among the individual N stages themselves, including N0 cases.

A similar trend was observed with respect to histological grading. Regardless of tumor grade, all tumor subgroups exhibited higher BSG expression levels compared to normal tissue (all *p* < 0.001), reflecting once again a general upregulation of BSG in malignant lesions (Figure 4D). Among intra-tumoral comparisons, a statistically significant increase in BSG expression was detected in grade 3 tumors compared to grade 1 (*p* = 0.038). In contrast, no other pairwise difference among tumor grades reached statistical significance.

**Figure 5:**
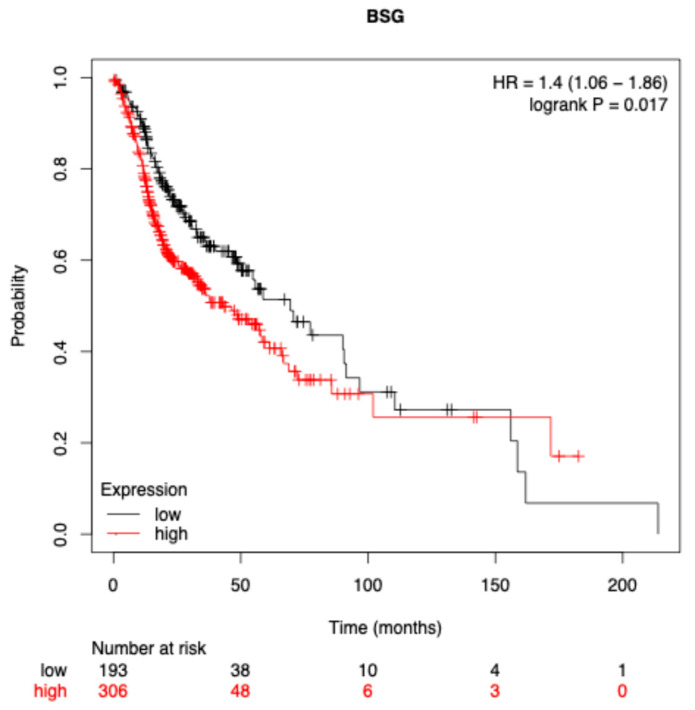
Kaplan–Meier survival analysis for overall survival in TCGA-HNSCC patients stratified by BSG (CD147) mRNA expression. Patients with high BSG expression showed significantly reduced overall survival compared to those with low expression (log-rank *p* = 0.017, HR = 1.40, 95% CI: 1.06–1.86). Plot exported from KMplot.com. Data accessed on 25 October 2025.

To assess the prognostic significance of BSG expression in HNSCC, a Kaplan–Meier survival analysis was performed using the KMplot.com tool, based on TCGA patient data. Patients were stratified into high- and low-expression groups based on the median BSG mRNA level. The analyses revealed a statistically significant difference in OS between the groups, with high BSG expression associated with reduced survival probability (log-rank *p* = 0.017, Figure 5). The calculated hazard ratio (HR) was 1.40 with a 95% CI of 1.06–1.86.

Taken together, these data indicate that BSG is significantly overexpressed in HNSCC and is associated with unfavorable clinical features and reduced overall survival.

#### 2.5.2. BSG-Linked Immune Modulation in the HNSCC Tumor Microenvironment

**Figure 6:**
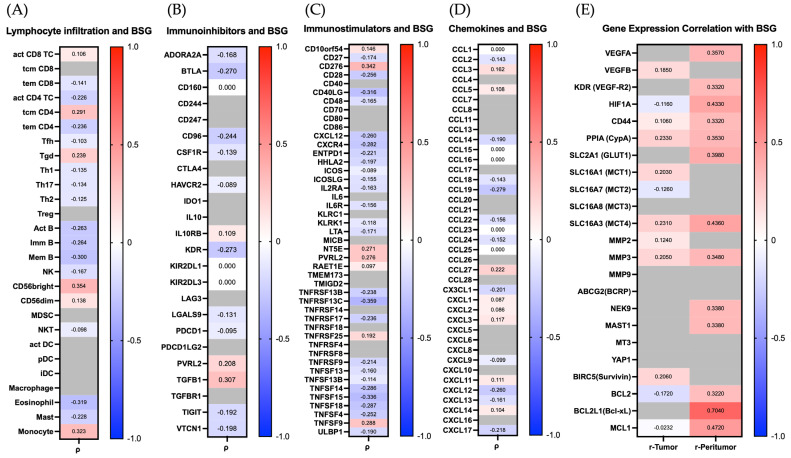
Correlation of BSG (CD147) expression with immunological and tumor-promoting gene signatures in TCGA-HNSCC cohorts (TISIDB, *n* = 522; GEPIA3, *n* = 520 tumors/44 normal tissues). Panel (**A**) shows correlations between BSG expression and lymphocyte infiltration across 27 immune cell types. Panel (**B**) displays associations with immunoinhibitory checkpoint molecules, and panel (**C**) with immunostimulatory genes. Panel (**D**) presents correlations with chemokines relevant to immune cell trafficking. Panel (**E**) depicts correlations between BSG expression and selected genes involved in tumor progression, metabolism, angiogenesis, and therapy resistance. Spearman’s *ρ* values are shown for panels (**A**–**D**) (TISIDB), and Pearson’s *r* values for panel (**E**) (GEPIA3). Red and blue colors indicate positive and negative correlations, respectively. Gray-shaded fields represent non-significant results (*p* ≥ 0.05). Data accessed on 25 October 2025.

To evaluate potential immunological functions of CD147 in the tumor microenvironment, correlation analyses were conducted using the TISIDB database (Figure 6).

Regarding lymphocyte infiltration, BSG expression showed consistently negative correlations with multiple T cell subtypes, including activated CD4^+^ T cells (*ρ* = −0.226), memory CD4^+^ (*ρ* = −0.236) and CD8^+^ (*ρ* = −0.141) T cells, memory B cells (*ρ* = −0.300), and eosinophils (*ρ* = −0.319) (Figure 6A). These associations may reflect reduced adaptive immune response in BSG-high tumors. Interestingly, a weak positive correlation was observed between BSG and CD56^++^ (bright) Natural Killer (NK) cells (*ρ* = 0.354) and Monocytes (*ρ* = 0.323), suggesting a potential compensatory innate immune component.

In terms of immune checkpoint interactions, BSG expression was positively correlated with the immunoinhibitory gene TGFB1 (*ρ* = 0.307). At the same time, weak inverse associations were observed for KDR (*ρ* = −0.273), BTLA (*ρ* = −0.270), and CD96 (*ρ* = −0.244), as shown in Figure 6B. Among immunostimulatory molecules, both positive and negative associations were detected. Notably, BSG expression showed a strong positive correlation with CD276 (*ρ* = 0.342), as well as a correlation with TNFSF9 (*ρ* = 0.288), while inverse correlations were observed with TNFRSF13C (*ρ* = −0.359), TNFRSF15 (*ρ* = −0.336), CD40LG (*ρ* = −0.316), and TNFSF14 (*ρ* = −0.286). These findings suggest that higher BSG expression may be associated with reduced immune co-stimulatory signaling (Figure 6C).

Finally, the analysis of chemokine expression revealed modest negative correlations between BSG and several chemokines involved in leukocyte recruitment, including CCL19 (*ρ* = −0.27.9), CXCL12 (*ρ* = −0.260), CX3CL1 (*ρ* = −0.201), and CCL18 (*ρ* = −0.143), suggesting a dampened chemotactic gradient in BSG-overexpressing tumors (Figure 6D).

In summary, high BSG expression appears to be associated with an immunosuppressive tumor microenvironment characterized by reduced lymphocyte infiltration and altered immune-modulatory signaling.

#### 2.5.3. Transcriptomic Correlation of CD147 with Key Regulator Genes of Tumor Promotion and Therapy Resistance in HNSCC

To deepen understanding of the molecular context surrounding CD147 expression and therapy resistance in OSCC, additional in silico correlation analyses were conducted using the GEPIA3 platform with HNSCC cohorts. Pearson correlation coefficients (*r*) were calculated between BSG and a selected panel of genes functionally implicated in tumor progression, immune regulation, and metabolic adaptation. As visualized in the correlation heatmap (Figure 6E), multiple genes showed considerable positive correlations with BSG expression.

In the peritumoral compartment, strong positive correlations were observed between BSG expression and several central effectors of oncogenic signaling and treatment resistance. These included HIF1A (HIF-1α; *r* = 0.433), SLC2A1 (GLUT1; *r* = 0.398), PPIA (CypA; *r* = 0.353), CD44 (*r* = 0.332), and SLC16A3 (MCT4; *r* = 0.436). These findings suggest a pattern that aligns with CD147’s known role in sustaining the glycolytic phenotype and facilitating lactate export. Furthermore, correlations with MMP3 (*r* = 0.348) and VEGFA (*r* = 0.357) support the involvement of BSG in extracellular matrix remodeling and angiogenesis.

Additionally, the anti-apoptotic regulator BCL2L1, coding for Bcl-xL, showed the strongest correlation (*r* = 0.704), along with MCL1 (*r* = 0.472) and BCL2 (*r* = 0.322), underscoring a potential link between CD147 and apoptosis resistance in the peritumoral niche, as these genes are critically involved in cell survival under therapeutic stress.

In contrast, correlations within the tumor core were generally weaker and more variable. Moderate associations were observed for PPIA (CypA; *r* = 0.233), MCT4 (*r* = 0.231), and BIRC5 (Survivin; *r* = 0.206). Negative correlations (HIF1A, SLC16A7, BCL2, and MCL1) are shown in blue shades, while non-significant correlations are covered in gray (VEGFA, KDR, SLC2A1, SLC16A8, MMP9, ABCG2, NEK9, MAST1, MT3, YAP1, and BCL2L1). Likewise, in the peritumoral compartment, non-significant results of VEGFB, SLC16A1, SLC16A7, SLC16A8, MMP2, MMP9, ABCG2, MT3, YAP1, and BIRC5 are covered in gray.

Taken together, this extended in silico analysis provides further evidence for the integration of CD147 into a pro-oncogenic transcriptional network that promotes tumor cell survival, metabolic adaptation, matrix degradation, and resistance to therapy. Notably, the strongest associations were observed in the peritumoral compartment, suggesting this zone as a critical interface for CD147-driven tumor-host interactions.

## 3. Discussion

In line with the growing interest in the metabolic and immunological landscape of solid tumors, this study investigated the expression patterns and clinical relevance of CD147 in oral squamous cell carcinoma (OSCC), focusing on its compartment-specific prognostic and predictive value in a large, well-characterized patient cohort.

Our findings indicate that low CD147 expression in the tumor periphery is associated with significantly prolonged overall survival, whereas CD147 levels in the tumor center or the adjacent mucosa showed no prognostic relevance. Such a pattern aligns with known pathophysiological processes, given that the invasive tumor front serves as the interface for tumor–stroma interactions, immune evasion, and metastatic potential [24,25]. Given the limited number of clinically implemented prognostic biomarkers in OSCC, the identification of CD147 as a compartment-specific prognostic indicator offers a promising addition to the current stratification tools. Compared to broadly expressed markers such as Epithelial-Growth-Factor-Receptor (EGFR), p53, or Survivin, CD147 may provide distinct spatial and functional information relevant to invasion dynamics and immune evasion [26].

This effect was confirmed in multivariate analysis, highlighting peripheral CD147 as an independent prognostic marker in OSCC. However, no statistically significant associations between CD147 expression and adjuvant therapy response were found across the examined compartments.

Multiple previous studies have demonstrated that high CD147 expression is associated with poorer survival outcomes across various cancer types [11,27,28]. Among others, one key mechanistic factor is its known role in activating the GLUT1-monocarboxylate transporter (MCT) axis, promoting glucose uptake and lactate efflux [13], and thereby supporting the Warburg metabolism and pH homeostasis in cancer [14]. CD147 also contributes to matrix metalloproteinase (MMP)-mediated extracellular matrix remodeling [29], angiogenesis [15], and invasion [30], which are critical mechanisms for tumor progression and metastasis.

In breast cancer, for example, Liu et al. linked elevated CD147 expression to chemoresistance and reduced therapy efficacy [16]. Similar findings have been reported in cervical and pancreatic cancer, supporting the hypothesis that CD147 confers therapy resistance by stabilizing survival pathways, inhibiting apoptosis, and modulating the tumor microenvironment [20,31].

Despite the biological rationale, we did not observe a significant association between CD147 expression and response to adjuvant therapy, including adjuvant treatment in general, radiotherapy, and radiochemotherapy. Although patients with low peripheral CD147 expression showed favorable trends, these did not reach statistical significance, likely due to small sample sizes within therapy subgroups. Consistent with this, logistic regression models demonstrated poor predictive accuracy, as shown by wide confidence intervals and a weak model fit.

In silico immunological and transcriptomic analyses of The Cancer Genome Atlas (TCGA) data, with a focus on head and neck squamous cell carcinoma (HNSCC), supported our tissue-based findings. Since most of these TCGA-based analyses are derived largely from bulk RNA sequencing, it should be emphasized that they cannot resolve intratumoral spatial heterogeneity and therefore should not be interpreted as a direct spatial validation of the compartment-specific IHC patterns observed in our cohort. Notably, only the GEPIA3 correlation analysis shown in Figure 6E provides a limited compartmental approximation by reporting correlations separately for tumor tissue and the GEPIA3-defined peritumoral tumor infiltration zone, whereas the remaining TCGA-derived outputs reflect aggregated bulk signals from the sampled tumor tissue. Within this framework, CD147 (Basigin, BSG) expression was significantly upregulated in tumor tissue and linked to decreased overall survival. The correlation with immune signatures displayed a complex pattern, with negative correlations with adaptive immune cells (e.g., CD8^+^ T cells, memory B cells) and positive associations with immunoinhibitory molecules (e.g., TGFB1, CD276), indicating a suppressive immune microenvironment in CD147-high tumors [32,33]. Interestingly, positive correlations with monocytes and CD56^bright^ natural killer (NK) cells were observed. This may reflect an innate compensatory immune response aimed at counterbalancing the immunosuppressive and tumor-promoting effects of CD147 overexpression, as similar mechanisms of blocking immunosuppression are the aim of several treatment modalities [34]. Given that CD56^bright^ NKs are typically less cytotoxic but highly cytokine-productive, their presence may also indicate an altered immune contexture with unresolved immunoregulatory signaling rather than effective tumor surveillance [35].

Also contributing to immune modulation, metabolic rewiring toward glycolysis with enhanced lactate export can acidify the local milieu and impair effector immune cell function, while supporting immunosuppressive phenotypes through metabolic–epigenetic regulation [36]. In OSCC, spatial and single-cell analyses indicate that hypermetabolic, acidified regions can foster chemokine-driven recruitment of regulatory T cells with increased TGF-β signaling, thereby shaping an immunosuppressive niche [37]. In parallel, CD147-driven matrix remodeling and protease activity can contribute to immune cell exclusion by restructuring extracellular barriers and altering stromal signaling [38,39]. Finally, CD147-related tumor-myeloid interactions have been mechanistically linked to macrophage recruitment and immunosuppression in other solid squamous tumors, supporting the plausibility of CD147-mediated immune-stroma cross-talk in squamous carcinomas [40]. Together, these lines of evidence provide a framework in which CD147-high tumors couple metabolic adaptation and invasive remodeling with immunoregulatory signaling, consistent with the immune correlations observed in our TCGA-based analyses.

Recent work further strengthens this immunometabolic interpretation by highlighting lactate as a signaling metabolite that actively shapes tumor-immune interactions and immunotherapy responsiveness, including through lactate-driven immunosuppression and lactylation-associated reprogramming of the tumor microenvironment [41]. In parallel, contemporary reviews of immunotherapy in head and neck cancer emphasize both the clinical relevance of immune checkpoint blockade and the need for mechanistically informed biomarkers and combination strategies to overcome immunosuppressive niches [42]. In this context, CD147’s functional coupling to lactate transport via MCTs and its association with immunoinhibitory signaling provide a plausible mechanistic link between a CD147-high, lactate-shaped microenvironment and the immune signatures observed in our TCGA-based analyses.

Transcriptomic correlation analyses showed that high BSG expression is part of a transcriptional network promoting oncogenic signaling, metabolic adaptation, and resistance to apoptosis. Therein, strong positive correlations were observed with key regulators, including HIF1A (HIF-1α), SLC2A1 (GLUT1), SLC16A3 (MCT4), and CD44, highlighting CD147’s role in maintaining a hypoxia-driven, glycolytic phenotype and in facilitating remodeling of the acidic microenvironment, which are hallmarks of aggressive tumor behavior [12,43,44]. Collectively, these correlations delineate a CD147-associated program that couples metabolic adaptation to microenvironmental remodeling. Hypoxia-driven glycolysis with enhanced lactate export can sustain tumor cell fitness under nutrient and oxygen limitation and simultaneously promote an acidic niche that favors invasion and stress tolerance. In parallel, CD147-linked extracellular matrix remodeling and invasion-associated proteolysis, including MMP-related signatures and urokinase-type plasminogen activator (uPA) pathway activity, provide a direct mechanistic route to basement membrane degradation, resulting in stromal invasion and regional spread in oral and head and neck squamous cell carcinoma models [38,39]. Importantly, this pathway becomes even clearer when tumors are compared with high versus low CD147 activity. In the CD147 high setting, the transcriptomic network converges on coordinated programs that are each directly relevant to OSCC progression, like afore-mentioned metabolic reprogramming under hypoxia (HIF1A, GLUT1, MCT4), remodeling of the acidic microenvironment, extracellular matrix degradation, and motility that enable invasion, and survival signaling consistent with reduced apoptosis. In contrast, CD147 low tumors are characterized by the relative attenuation of these coupled stress-adaptation and remodeling modules and, as presented in our TCGA-based immune correlation analysis, a comparatively less suppressive immune contexture, as reflected by inverse associations of BSG with adaptive immune cell signatures and positive associations with immunoinhibitory mediators in CD147 high tumors. Taken together, these patterns support a model in which high CD147 expression promotes a progression-prone phenotype by integrating metabolic adaptation, invasive remodeling, and immune suppression, whereas lower CD147 expression aligns with a less aggressive transcriptional and microenvironmental state.

Notably, high co-expression with anti-apoptotic genes such as BCL2L1 (Bcl-xL), MCL1, MAST1, and BCL2 further supports CD147’s role in treatment resistance and cell survival under stress conditions, such as radiation or chemotherapy [17,31,45,46]. These correlations were strongest in the peritumoral compartment, highlighting this area as a potential hotspot for CD147-mediated tumor-host interactions. The lack of similar correlation strength in the tumor core suggests spatially limited relevance, consistent with our IHC-based findings.

Importantly, the mechanistic pathway programs linked to high BSG expression are further substantiated by meta-analyses and evidence from larger cohorts, which consistently associate key nodes of this network with prognosis and survival, thereby strengthening the translational bridge from transcriptomic signatures to clinically observable outcomes. Consistent with this concept, pooled OSCC evidence shows that HIF-1α overexpression is associated with adverse clinicopathological features and significantly worse overall survival, in line with a hypoxia-adapted, therapy-resistant tumor state [43]. Likewise, GLUT1 overexpression has been linked to aggressive disease characteristics and shorter overall survival in OSCC, and broader meta-analytic evidence across HNSCC supports the notion that glycolysis markers, including GLUT1 and MCT4, are associated with inferior survival outcomes [47,48]. Along the same line, high MCT4 expression has been reported to correlate with poor prognosis in OSCC cohorts, aligning with the concept that lactate export and extracellular acidification foster invasion, immune evasion, and treatment resistance. Beyond metabolism, progression-associated proteolysis programs that converge on uPA and MMP activity have been shown to predict metastatic risk and unfavorable prognosis in OSCC, supporting the interpretation that the BSG-correlated invasion signature reflects clinically meaningful aggressiveness [49,50,51,52]. Taken together, these external prognostic data provide a plausible bridge between the transcriptomic alterations observed in the context of high CD147/BSG expression and OSCC progression.

From a translational perspective, targeting CD147 directly or disrupting its interaction partners (e.g., MCTs, CD44, or cyclophilin A) could represent a viable therapeutic strategy, particularly in tumors displaying high peripheral CD147 expression. Direct CD147-targeting modalities (e.g., functional antibodies and emerging antibody-based formats such as antibody–drug conjugates in other tumor entities) provide proof-of-principle that CD147 is a druggable surface antigen [53]. Mechanistically, additional rational strategies include disrupting the CD147–cyclophilin A axis and targeting lactate transport/metabolic dependencies that shape the local microenvironment, as MCT-focused approaches are being evaluated clinically in advanced cancers in phase I trials [54]. In head-and-neck models, combined targeting of CD147/EMMPRIN and EGFR has shown additive inhibition of proliferation and migration compared with either approach alone, supporting combination concepts aligned with current multimodal care [55]. Furthermore, preclinical models in other tumor types have demonstrated the efficacy of CD147-targeting small molecules, underscoring CD147 as a potential therapeutic target in OSCC [56].

Despite the comprehensive design and the integration of clinical, immunohistochemical, and transcriptomic data, several limitations should be acknowledged. Given the retrospective, monocentric nature of the study, the scope of the conclusions should be interpreted in the context of the study design. This cohort comprises retrospectively collected OSCC patients treated at a single tertiary care center over a defined period, and thus, the observed effect sizes may be influenced by center-specific referral patterns, case mix, and local treatment algorithms. Moreover, because all patients were from a single institution and a relatively homogeneous population, extrapolation to other geographic regions, ethnic backgrounds, and contemporary treatment settings should be done with caution. Moreover, the incomplete granularity of some clinical variables limits causal inference and may lead to residual confounding. In addition, the TMA-based approach, while enabling standardized high-throughput assessment, samples only selected cores and may therefore not fully capture intratumoral heterogeneity, particularly across the invasive front and the tumor–stroma interface. Likewise, immunohistochemical scoring was semi-quantitative and may be susceptible to interobserver variability. Although efforts were made to standardize scoring, digital quantification may further enhance reproducibility. In this context, multiplex fluorescence approaches combined with digital analysis could also enable a more detailed spatial assessment of immune infiltration in relation to CD147 expression, and work in this direction is ongoing. Furthermore, treatment-response analyses, especially for radiochemotherapy, were constrained by small subgroup sizes, which reduced statistical power and may have left clinically meaningful trends underpowered. At the same time, the transcriptomic analyses presented here are primarily correlation-based and therefore describe co-expression networks rather than mechanistic causality. Also, functional validation experiments were not performed to confirm the biological roles of CD147 and its correlated partners. Finally, although TCGA-HNSCC data provided valuable external context, its anatomical heterogeneity prevents definitive OSCC-specific generalization.

To overcome these limitations and expand on the presented findings, the following future directions are either considered or already underway: A prospective, multicenter validation study represents a logical next step to confirm the prognostic value of CD147 expression in OSCC. Such a study, while not yet initiated, would ideally include standardized digital immunoscoring and molecular stratification to enhance external validity and clinical translatability. Functional in vitro studies are currently in preparation to investigate the causal role of CD147 in proliferation, apoptosis resistance, and treatment response. Planned approaches include CRISPR/Cas9-mediated gene editing in OSCC cell lines and co-culture experiments with stromal and immune components to explore CD147-dependent tumor-host interactions. Fluorescence-based multichannel imaging analyses are already in progress. These aim to quantitatively assess spatial co-localization between CD147 and immune cell infiltrates in TMA sections, again focusing on the tumor invasive front. This method is expected to yield high-resolution insight into the immunological landscape shaped by CD147 expression in the tumor microenvironment.

## 4. Materials and Methods

### 4.1. Patient Cohort and Clinical Data

As already described by Erber et al., oral squamous cell carcinoma (OSCC) specimens from 229 patients were collected at the University Hospital Regensburg, in the Cranio-Maxillofacial Surgery Department between 2003 and 2014 [57]. All 229 patients involved in this study were Caucasian and received primary surgical treatment with additional neck dissection as recommended by radiological and clinical findings. No neoadjuvant therapy or prior neck dissections were carried out beforehand. The tumor samples included were declared R0 by a pathologist at the Institute of Pathology, University Hospital Regensburg. Tumor staging was performed as recommended by the Union Internationale Contre le Cancer (UICC) and their guidelines in the 8th edition [58]. Medical records were retrospectively reviewed to gather clinical data. Gender, age at diagnosis, nicotine abuse, and alcohol abuse were extracted from medical history forms. The anatomical region of resected tumors, T-status, Grading, N-status, UICC stage, lymph vessel invasion, vessel invasion, and perineural invasion were gathered from the final respective histopathological reports. Survival, recurrence, and adjuvant therapy were calculated and logged from tumor follow-up protocols and personal telephone inquiries.

### 4.2. Tissue Microarray (TMA), Immunohistochemistry (IHC), Image Analysis, and Scoring

Tumor samples were formalin-fixed, paraffin-embedded, routinely processed, and assembled into a tissue microarray as previously described [57,59]. In short, for each patient, three tissue cores were obtained through punch biopsy with a diameter of 2 mm. Two cores were taken separately from the resected tumor sample: one from the tumor center and the other from the peripheral invasive front. Additionally, the adjacent mucosal core was not obtained from the tumor resection blocks but from a separate biopsy of the same patient’s clinically uninvolved oral mucosa on the contralateral side of the oral cavity. These three tissue cores from each patient were embedded into paraffin microarray blocks, with cores from 20 patients per block. The blocks were subsequently cut into 2 µm thick sections, which were mounted on microscope slides for immunohistochemical staining.

**Figure 7:**
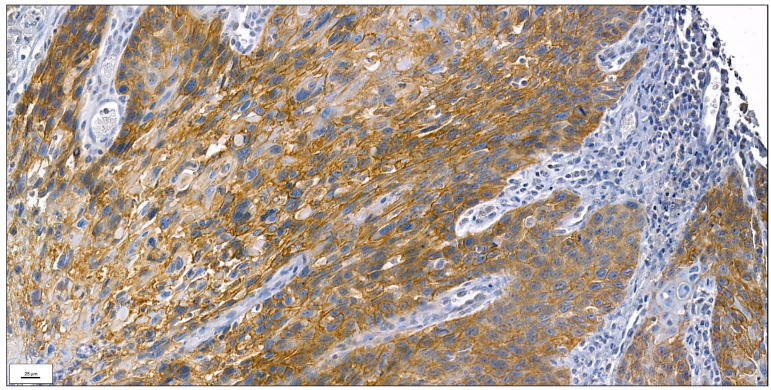
Representative immunohistochemical staining of CD147 in oral squamous cell carcinoma showing predominantly membranous expression in neoplastic squamous cells. Original magnification ×400. CD147 immunoreactivity was visualized with DAB (brown) and nuclei were counterstained with hematoxylin (blue).

Immunohistochemistry for CD147 was performed on these TMA sections. Staining was carried out manually using a mouse monoclonal anti-CD147 antibody (clone: EMMPRIN (8D6), sc-21746; Santa Cruz Biotechnology, Heidelberg, Germany; dilution 1:50), with an incubation time of 30 min at room temperature, followed by DAB visualization and hematoxylin counterstaining (representative staining shown in Figure 7).

CD147-stained TMA slides were scanned using the Pannoramic 1000 scanner (3DHistech, Budapest, Hungary) and visualized with CaseViewer software (Version 2.4; 3DHistech, Budapest, Hungary). Scanning was performed analogously to the workflow described by Eichberger et al. [60]. Image evaluation was conducted by a board-certified pathologist (R.E.), blinded to all clinicopathological and outcome data.

Corresponding H&E sections, prepared according to the standard in-house protocol, were first reviewed to confirm tissue quality, morphology, and tumor growth pattern in OSCC. To ensure consistent spatial annotation despite the inherent sampling limitations of a TMA approach, each stained TMA core was then re-reviewed during digital slide evaluation and compared to the corresponding H&E section to confirm tissue integrity and correct compartment allocation. Cores were considered evaluable only if they contained sufficient, well-preserved target tissue for reliable scoring (viable tumor for tumor compartments, intact epithelium for mucosa) with adequate morphology and without dominant artefacts. Cores were marked as missing tissue if the tissue cylinder was absent or mostly lost during sectioning or staining. Cores were deemed non-evaluable if present but unsuitable for assessment due to extensive folding, crush, or cautery artefacts, necrosis, limited target tissue, or unclear compartment representation. Non-evaluable or missing cores were excluded from the respective compartment-specific immunoscore analyses.

CD147 expression was then semiquantitatively assessed for each TMA core. Evaluation was based on staining intensity (graded 0–3) and the percentage of positively membranous stained tumor cells (0–100%). An “immunoscore” was calculated by multiplying intensity and percentage values, yielding a score range of 0–300. CD147 expression was categorized as negative (0), low (1–20), or high (>20) [16]. In addition, CD147 expression was evaluated separately in intraepithelial tumor areas and stromal compartments.

### 4.3. Statistical Analysis

All statistical analyses were performed using IBM SPSS Statistics version 29.0.0 (IBM Corp., Armonk, NY, USA). A *p*-value < 0.05 was considered statistically significant for all applied tests.

Descriptive statistics were used to summarize clinicopathological characteristics. Further, to explore potential associations between CD147 immunoscore levels (negative, low, and high) and clinicopathological parameters, nonparametric statistical tests were applied due to the non-normally distributed and ordinal nature of the immunoscore data. Differences in CD147 expression between two groups were evaluated using the Mann–Whitney U test. Comparisons among multiple ordered categories were analyzed using the Kruskal–Wallis test. For variables with a natural order, Jonckheere-Terpstra tests were applied to identify trend associations. The Jonckheere-Terpstra and Mann–Whitney U tests were standardized to a z-score, and two-tailed asymptotic significance levels were reported. In significant Kruskal–Wallis test results, pairwise post hoc comparisons were conducted using Bonferroni correction to identify specific group differences.

Overall survival (OS) was analyzed using the Kaplan–Meier method to estimate survival probabilities and to compare survival distributions between CD147 immunoscore groups (negative, low, and high) within the tumor center, tumor periphery, and adjacent mucosa. The time-to-event variable was defined as the period between initial tumor resection and death (event). Patients who were alive at the last follow-up were treated as censored cases, represented by cross marks (+) in the survival plots. Differences in survival distributions were assessed using the Mantel–Cox log-rank test. 

To complement Kaplan–Meier analysis, univariate and multivariate Cox proportional hazards regression analyses were conducted to evaluate prognostic factors for overall survival. Variables with *p*-value < 0.10 in univariate analysis were included in the multivariate model. The proportional hazards assumption was verified by log-minus-log plots (plots not included). Hazard ratios (HRs) and 95% confidence intervals (CIs) were calculated for each variable. Model calibration and goodness-of-fit were assessed using the Hosmer-Lemeshow test, and model discrimination was evaluated using classification accuracy and residual inspection.

For patients receiving adjuvant treatment, binary logistic regression analyses were performed across tissue compartments to evaluate treatment response. Patients who did not receive adjuvant treatment were excluded from these analyses. CD147 immunoscore in relevant tissue compartments was defined as a categorical independent variable using indicator coding, with the negative expression group serving as the reference category. The dependent variable was the dichotomized treatment outcome, defined as success (survival) or failure (death) at the time of last follow-up. Analyses were performed for adjuvant treatment in general, radiation therapy, and radiochemotherapy. The Enter method was used for model estimation. Odds ratios (ORs) with 95% confidence intervals (CIs) were calculated for each CD147 category. Model calibration was assessed with the Hosmer-Lemeshow goodness-of-fit test. The overall classification accuracy was evaluated by comparing the predicted and observed outcomes using residual analysis. Cox & Snell and Nagelkerke R^2^ coefficients were summarized to assess model performance.

Stratified subgroup survival analyses and therapy-response logistic regression models were conducted in an exploratory manner; no formal adjustment for multiple testing was applied, and results are interpreted as hypothesis-generating.

### 4.4. Bioinformatic Analysis of BSG Expression Correlations and Immunological Associations in the Cancer Genome Atlas (TCGA)

Gene expression and clinical data for head and neck squamous cell carcinoma (HNSCC) were analyzed using publicly accessible online platforms. The expression of the Basigin (BSG) gene (encoding CD147) was evaluated using RNA-sequencing data from TCGA and the University of Alabama at Birmingham Cancer data analysis (UALCAN) web portal (http://ualcan.path.uab.edu, last accessed on 25 October 2025). Analyses included comparisons of BSG expression between primary tumors (*n* = 520) and normal tissues (*n* = 44), as well as subgroup analyses by lymph node metastasis status (N0–N3), histological grade (G1–G3), and tumor stage (UICC stage I–IV). Statistical comparisons of gene expression levels between groups were performed using unpaired Student’s *t*-tests as implemented by the UALCAN platform.

Overall survival (OS) analysis was performed using the Kaplan–Meier Plotter tool (http://KMplot.com, last accessed on 25 October 2025), which stratifies 499 HNSCC patients from the TCGA data pool by median BSG expression and estimates survival using the Kaplan–Meier method with log-rank test significance.

To explore the relationships between BSG expression and the tumor immune microenvironment, the TISIDB database (http://cis.hku.hk/TISIDB/index.php, last accessed on 25 October 2025) was utilized. Correlation analyses were conducted to examine BSG expression in relation to immune cell infiltration levels, immune-stimulatory and inhibitory checkpoint molecules, as well as chemokines and their receptors in 522 patients with HNSCC. Spearman correlation coefficients (*ρ*) were calculated, and *ρ* values ≥ 0.3 and *p* < 0.05 were considered statistically significant and biologically relevant. In addition, to further investigate potential transcriptional associations of BSG expression in HNSCC, gene expression profiling was conducted using the GEPIA 3 online platform (https://gepia3.bioinfoliu.com, last accessed on 25 October 2025), which integrates TCGA and the Genotype-Tissue Expression (GTEx) project’s datasets. A targeted correlation analysis was performed to identify genes that showed significant co-expression with BSG across the HNSCC cohort, both in the tumor and the tumor infiltration zone, as described by GEPIA3 as the peritumor region in 520 and 44 cases of HNSCC, respectively. Pearson correlation coefficients (*r*) were calculated between BSG and selected genes of interest, including those involved in metabolism, matrix remodeling, immune modulation, and hypoxia-related pathways. In these correlation analyses, *r* values ≥0.3 and *p* < 0.05 were considered significantly correlated and relevant. The results of BSG expression correlations are displayed in heatmaps.

All databases were last accessed on 25 October 2025. The Kaplan–Meier survival curve was exported from http://KMplot.com. Figures, including plots and heatmaps, were generated using GraphPad Prism 10 (version 10.6.1; GraphPad Software, San Diego, CA, USA).

## 5. Conclusions

This study demonstrates that CD147 expression in oral squamous cell carcinoma is spatially heterogeneous and carries distinct prognostic implications depending on the tumor compartment. Low CD147 expression at the invasive tumor front was independently associated with improved overall survival, underscoring the prognostic relevance of this tumor–stroma interface. In contrast, CD147 expression in the tumor center and adjacent mucosa showed no predictive value, and no significant associations with therapy response were identified.

These findings highlight the potential of compartment-specific CD147 expression as a prognostic biomarker in OSCC and lay the groundwork for future studies targeting CD147-related pathways, as directed by TCGA data. External validation, functional in vitro analyses, and prospective multicenter studies are warranted to elucidate its biological role and translational relevance in tumor progression, immune evasion, and treatment resistance.

## Figures and Tables

**Table 1:** Baseline of Clinicopathological Features, UICC 8. Edition, *n* = 229.

Variable	Category	*n*	%
Immunoscore level tumor-center	Negative	33	14.4
	Low	52	22.7
	High	117	51.1
Immunoscore level tumor-periphery	Negative	34	14.8
	Low	37	16.2
	High	112	48.9
Immunoscore level mucous membrane	Negative	54	23.6
	Low	71	31.0
	High	1	0.4
Gender	Female	66	28.8
	Male	163	71.2
Age at diagnosis	<70	179	78.2
	≥70	50	21.8
Nicotine abuse	No	50	21.8
	Yes	179	78.2
Alcohol abuse	No	67	29.3
	Yes	162	70.7
Anatomical region	Floor of the mouth	108	47.2
	Tongue	26	11.4
	Hard palate	8	3.5
	Upper alveolar ridge/gingiva	12	5.2
	Lower alveolar ridge/gingiva	49	21.4
	Buccal mucous membrane	26	11.4
T-status	T1	72	31.4
	T2	79	34.5
	T3	15	6.6
	T4	63	27.5
	T1/T2	151	65.9
	T3/T4	78	34.1
Grading	G1	14	6.1
	G2	190	83.0
	G3	25	10.9
N-Status	N−	127	55.5
	N+	102	44.5
UICC-stage	I	51	22.3
	II	40	17.5
	III	32	14.0
	IV	106	46.3
	I/II	91	39.7
	III/IV	138	60.3
Recurrence	No	171	74.7
	Yes	58	25.3
Lymph vessel invasion	L0	188	82.1
	L1	41	17.9
Vessel invasion	V0	219	95.6
	V1	10	4.4
Perineural invasion	Pn0	222	96.6
	Pn1	7	3.1
Adjuvant therapy	No	109	47.6
	Radiotherapy	86	37.6
	Radiochemotherapy	34	14.8
Death	No	79	34.5
	Yes	150	65.5

Note: To facilitate direct comparison, subgroup classifications were created for the clinicopathological variables T-status and UICC-stage: T1 and T2 tumors were combined (T1/T2) and contrasted with advanced tumors T3 and T4 (T3/T4). Similarly, UICC-stages I and II were grouped together (I/II) and compared with the more advanced stages III and IV (III/IV).

**Table 2:** Association of CD147 immunoscore (tumor center) with clinicopathological parameters, UICC 8. Edition, *n* = 202.

Variable	Category	*n*	Test	*p*
Gender	Female/Male	59/143	Mann–Whitney-U	0.587
Age at diagnosis	<70/≥70	155/47	Mann–Whitney-U	0.608
T-status	T1/T2/T3/T4	59/73/13/57	Kruskal–Wallis	0.584
T-status	(T1/T2) and (T3/T4)	132/70	Jonckheere-Terpstra	0.064
Grading	G1/G2/G3	12/168/22	Kruskal–Wallis	0.571
N-Status	N−/N+	93/109	Mann–Whitney-U	0.776
UICC-stage	I/II/III/IV	41/36/30/95	Kruskal–Wallis	0.607
UICC-stage	(I/II) and (III/IV)	81/121	Jonckheere-Terpstra	0.850
Recurrence	No/Yes	149/53	Mann–Whitney-U	0.671
Lymph vessel invasion	L0/L1	163/39	Mann–Whitney-U	0.322
Vessel invasion	V0/V1	192/10	Mann–Whitney-U	0.062
Perineural invasion	Pn0/Pn1	195/7	Mann–Whitney-U	0.747
Adjuvant therapy	No/Radiotherapy/Radiochemotherapy	95/76/31	Kruskal–Wallis	0.732

Note: *p*-values were calculated using non-parametric tests as indicated (Mann–Whitney U, Kruskal–Wallis, Jonckheere–Terpstra). A *p*-value < 0.05 was considered statistically significant.

**Table 3:** Association of CD147 immunoscore (tumor periphery) with clinicopathological parameters, UICC 8. Edition, *n* = 183.

Variable	Category	*n*	Test	*p*
Gender	Female/Male	53/130	Mann–Whitney-U	0.907
Age at diagnosis	<70/≥70	140/43	Mann–Whitney-U	0.709
T-status	T1/T2/T3/T4	46/70/14/53	Kruskal–Wallis	0.354
T-status	(T1/T2) and (T3/T4)	122/61	Jonckheere-Terpstra	0.146
Grading	G1/G2/G3	10/153/20	Kruskal–Wallis	0.425
N-Status	N−/N+	87/96	Mann–Whitney-U	0.359
UICC-stage	I/II/III/IV	31/37/30/85	Kruskal–Wallis	0.264
UICC-stage	(I/II) and (III/IV)	75/108	Jonckheere-Terpstra	0.558
Recurrence	No/Yes	137/46	Mann–Whitney-U	0.816
Lymph vessel invasion	L0/L1	147/36	Mann–Whitney-U	0.707
Vessel invasion	V0/V1	174/9	Mann–Whitney-U	0.316
Perineural invasion	Pn0/Pn1	177/6	Mann–Whitney-U	0.212
Adjuvant therapy	No/Radiotherapy/Radiochemotherapy	80/74/29	Kruskal–Wallis	0.355

Note: *p*-values were calculated using non-parametric tests as indicated (Mann–Whitney U, Kruskal–Wallis, Jonckheere–Terpstra). A *p*-value < 0.05 was considered statistically significant.

**Table 4:** Association of CD147 immunoscore (adjacent mucosa) with clinicopathological parameters, UICC 8. Edition, *n* = 126.

Variable	Category	*n*	Test	*p*
Gender	Female/Male	37/89	Mann–Whitney-U	0.296
Age at diagnosis	<70/≥70	97/29	Mann–Whitney-U	0.026
T-status	T1/T2/T3/T4	36/47/9/34	Kruskal–Wallis	0.518
T-status	(T1/T2) and (T3/T4)	80/46	Jonckheere-Terpstra	0.580
Grading	G1/G2/G3	10/101/15	Kruskal–Wallis	0.335
N-Status	N−/N+	58/68	Mann–Whitney-U	0.515
UICC-stage	I/II/III/IV	24/25/17/60	Kruskal–Wallis	0.837
UICC-stage	(I/II) and (III/IV)	56/70	Jonckheere-Terpstra	0.246
Recurrence	No/Yes	93/33	Mann–Whitney-U	0.107
Lymph vessel invasion	L0/L1	101/25	Mann–Whitney-U	0.131
Vessel invasion	V0/V1	118/8	Mann–Whitney-U	0.659
Perineural invasion	Pn0/Pn1	130/6	Mann–Whitney-U	0.651
Adjuvant therapy	No/Radiotherapy/Radiochemotherapy	54/51/21	Kruskal–Wallis	0.960

Note: *p*-values were calculated using non-parametric tests as indicated (Mann–Whitney U, Kruskal–Wallis, Jonckheere–Terpstra). A *p*-value < 0.05 was considered statistically significant.

**Table 5:** Univariate and Multivariate Cox-Regression Analysis, UICC 8. Edition, *n* = 229.

Variable	Category	Univariate Cox Regression				Multivariate Cox Regression			
		*p*	HR	Lower 95% CI	Upper 95% CI	*p*	HR	Lower 95% CI	Upper 95% CI
Immunoscore level Tumor-center	Negative		1.000				-		
	Low	0.758	0.923	0.554	1.537	-	-	-	-
	High	0.366	0.811	0.516	1.277	-	-	-	-
Immunoscore level Tumor-periphery	Negative		1.000				1.000		
	Low	0.028	0.514	0.284	0.930	0.024	0.492	0.266	0.909
	High	0.775	0.775	0.598	1.466	0.568	0.872	0.545	1.394
Immunoscore level Mucous Membrane	Negative		1.000				-		
	Low	0.897	0.971	0.621	1.518	-	-	-	-
	High	0.971	0.964	0.131	7.079	-	-	-	-
Gender	Female		1.000				-		
	Male	0.575	1.109	0.772	1.593	-	-	-	-
Age at diagnosis	<70		1.000				1.000		
	≥70	0.002	1.778	1.235	2.560	0.001	2.036	1.322	3.135
Nicotine abuse	No		1.000				-		
	Yes	0.825	1.046	0.704	1.553	-	-	-	-
Alcohol abuse	No		1.000				-		
	Yes	0.984	1.004	0.704	1.430	-	-	-	-
Anatomical region	Buccal mucous membrane		1.000				-		
	Upper alveolar ridge/gingiva	0.551	1.295	0.553	3.032	-	-	-	-
	Lower alveolar ridge/gingiva	0.357	1.320	0.731	2.385	-	-	-	-
	Hard palate	0.727	0.823	0.275	2.464	-	-	-	-
	Tongue	0.633	0.844	0.421	1.692	-	-	-	-
	Floor of the mouth	0.586	0.859	0.498	1.482	-	-	-	-
T-status	T1		1.000				1.000		
	T2	0.111	1.396	0.926	2.103	0.899	0.951	0.440	2.056
	T3	0.061	1.893	0.971	3.690	0.103	2.163	0.855	5.476
	T4	0.001	2.028	1.324	3.106	0.294	1.606	0.663	3.889
Grading	G1		1.000				-		
	G2	0.493	1.285	0.628	2.630	-	-	-	-
	G3/G4	0.164	1.800	0.787	4.118	-	-	-	-
N-Status	N−		1.000				1.000		
	N+	0.004	1.601	1.161	2.207	0.020	2.253	1.134	4.475
UICC-stage	I		1.000				1.000		
	II	0.361	1.291	0.746	2.232	0.914	1.056	0.394	2.834
	III	0.048	1.735	1.004	3.000	0.149	0.459	0.159	1.323
	IV	0.007	1.822	1.175	2.826	0.252	0.523	0.172	1.585
Recurrence	No		1.000				1.000		
	Yes	0.004	1.675	1.181	2.375	0.021	1.613	1.074	2.423
Lymph vessel invasion	L0		1.000				1.000		
	L1	<0.001	1.958	1.321	2.901	0.009	1.946	1.179	3.213
Vessel invasion	V0		1.000				1.000		
	V1	0.020	2.234	1.135	4.399	0.729	1.152	0.517	2.565
Perineural invasion	Pn0		1.000				-		
	Pn1	0.968	1.021	0.377	2.761	-	-	-	-
Adjuvant therapy	No		1.000				1.000		
	Radiotherapy	0.018	1.520	1.074	2.152	0.549	1.160	0.714	1.884
	Radiochemotherapy	0.233	1.345	0.826	2.188	0.630	0.851	0.440	1.642

Note: Cox proportional hazards regression was used to assess the association between clinicopathological variables and overall survival. Variables with *p* < 0.10 in univariate analysis were included in the multivariate model. HR: hazard ratio; CI: confidence interval; -: variable not included in the multivariate model due to lack of significance in univariate analysis.

## Data Availability

Publicly available datasets were analyzed in this study. TCGA-HNSC RNA-seq and associated clinical data are available from the NCI Genomic Data Commons (GDC) Data Portal (https://portal.gdc.cancer.gov/; project: TCGA-HNSC). Additional expression, correlation, immune-signature, and survival analyses were performed using publicly accessible web resources, including GEPIA3 (https://gepia3.bioinfoliu.com; based on TCGA and GTEx), UALCAN (http://ualcan.path.uab.edu/), TISIDB (http://cis.hku.hk/TISIDB/index.php), and the Kaplan–Meier Plotter (http://KMplot.com), which provide open access to processed data derived primarily from TCGA. The immunohistochemistry immunoscores and de-identified clinicopathological annotations generated for the institutional OSCC cohort are available from the corresponding author upon reasonable request and subject to institutional and ethical approvals.

## Abbreviations

The following abbreviations are used in this manuscript:

OSCC	Oral Squamous Cell Carcinoma
HNSCC	Head and Neck Squamous Cell Carcinoma
CD147	Cluster of Differentiation 147
BSG	Basigin
EMMPRIN	Extracellular Matrix Metalloproteinase Inducer
IHC	Immunohistochemistry
TMA	Tissue Microarray
TCGA	The Cancer Genome Atlas
TISIDB	Tumor and Immune System Interaction Database
GEPIA	Gene Expression Profiling Interactive Analysis
GLUT1	Glucose Transporter 1
MCT	Monocarboxylate Transporter
HIF-1α	Hypoxia-Inducible Factor 1-alpha
MMP	Matrix Metalloproteinase
VEGF	Vascular Endothelial Growth Factor
NK cells	Natural Killer cells
HR	Hazard Ratio
CI	Confidence Interval
OS	Overall Survival
OR	Odds Ratio
EMT	Epithelial–Mesenchymal Transition
CRISPR	Clustered Regularly Interspaced Short Palindromic Repeats
CypA	Cyclophilin A
UALCAN	University of Alabama at Birmingham Cancer Data Analysis Portal
KMplot	Kaplan–Meier Plotter
SPSS	Statistical Package for the Social Sciences

## Appendix A

**Table A1:** Significant Kaplan–Meier survival analyses of overall survival according to CD147 immunoscore (negative, low, high), stratified by clinicopathological parameters and tissue compartments.

Clinicopathological Parameter	Subgroup	Tissue Compartment	*n*	χ^2^	df	Log-Rank *p*
Gender	Female/Male	Tumor periphery	183	6.769	2	0.034
Age at diagnosis	<70/≥70	Tumor periphery	183	6.540	2	0.038
Grading	G1	Tumor center	202	13.992	2	<0.001
	G2	Tumor center	202	0.633	2	0.729
	G3	Tumor center	202	0.571	2	0.752
Grading	G1	Tumor periphery	183	0.128	2	0.938
	G2	Tumor periphery	183	7.629	2	0.022
	G3	Tumor periphery	183	0.170	2	0.918
Recurrence	No/Yes	Tumor periphery	183	6.478	2	0.039
V-status	V0/V1	Tumor periphery	183	6.299	2	0.043

Abbreviations: χ^2^, chi-square statistic; df, degrees of freedom. Note: Kaplan–Meier survival analyses were performed using the Mantel–Cox log-rank test. Clinicopathological parameters and CD147 immunoscore compartments stratified results. A *p*-value < 0.05 was considered statistically significant; values between 0.05 and 0.10 were interpreted as statistical trends.

**Table A2:** Logistic Regression Analysis of Therapy Response to Adjuvant Therapy in General, Reference Category “high CD147 Immunoscore”, *n* = 107.

Region	Score Comparison	Odds Ratio (OR)	95% CI	*p*-Value	Model χ^2^	df	Model *p*-Value
Tumor Center	Negative vs. High	0.59	0.117–2.976	0.523	0.481	2	0.786
	Low vs. High	0.821	0.277–2.435	0.723			
Tumor Periphery	Negative vs. High	0.828	0.208–3.302	0.789	3.084	2	0.214
	Low vs. High	2.576	0.838–7.921	0.099			
Adjacent Mucosa	Negative vs. High	5.6 × 10^8^	n.e.	1.0	0.586	2	0.746
	Low vs. High	5.3 × 10^8^	n.e.	1.0			

Note: CD147 immunoscore expression was analyzed as a categorical variable (negative, low, high), with “high” serving as the reference category. Logistic regression models were computed separately for each tumor region. n.e. = not estimable due to insufficient case numbers in the “low” expression subgroup of the mucosal compartment.

**Table A3:** Logistic Regression Analysis of Therapy Response to Adjuvant Radiotherapy, Reference Category “high CD147 Immunoscore”, *n* = 76.

Region	Score Comparison	Odds Ratio (OR)	95% CI	*p*-Value	Model χ^2^	df	Model *p*-Value
Tumor Center	Negative vs. High	0.296	0.034–2.595	0.272	1.816	2	0.403
	Low vs. High	0.561	0.135–2.333	0.427			
Tumor Periphery	Negative vs. High	0.556	0.106–2.901	0.486	2.377	2	0.305
	Low vs. High	2.160	0.579–8.055	0.251			
Adjacent Mucosa	Negative vs. High	1.250	0.326–4.797	0.745	0.105	1	0.746
	Low vs. High	n.e.	n.e.	–			

Note: CD147 immunoscore expression was analyzed as a categorical variable (negative, low, high), with “high” serving as the reference category. Logistic regression models were computed separately for each tumor region. n.e. = not estimable due to insufficient case numbers in the “low” expression group of the mucosal compartment.

**Table A4:** Logistic Regression Analysis of Therapy Response to Adjuvant Radiochemotherapy, Reference Category “high CD147 Immunoscore”, *n* = 31.

Region	Score Comparison	Odds Ratio (OR)	95% CI	*p*-Value	Model χ^2^	df	Model *p*-Value
Tumor Center	Negative vs. High	4.667	0.223–97.497	0.321	1.005	2	0.605
	Low vs. High	1.556	0.256–9.469	0.632			
Tumor Periphery	Negative vs. High	3.000	0.203–44.359	0.424	1.795	2	0.408
	Low vs. High	4.000	0.458–34.922	0.210			
Adjacent Mucosa	Negative vs. High	6.92 × 10^8^	n.e.	1.000	1.056	2	0.590
	Low vs. High	1.08 × 10^9^	n.e.	1.000			

Note: CD147 immunoscore expression was analyzed as a categorical variable (negative, low, high), with “high” serving as the reference category. Logistic regression models were computed separately for each tumor region. n.e. = not estimable due to extreme OR values and limited sample sizes, particularly in the mucosal region.

## References

[1] Bray F., Ferlay J., Soerjomataram I., Siegel R.L., Torre L.A., Jemal A. (2018). Global cancer statistics 2018: GLOBOCAN estimates of incidence and mortality worldwide for 36 cancers in 185 countries. CA A Cancer J. Clin..

[2] Scully C., Bagan J. (2009). Oral squamous cell carcinoma overview. Oral Oncol..

[3] Chow L.Q.M. (2020). Head and Neck Cancer. N. Engl. J. Med..

[4] Hanahan D., Weinberg R.A. (2000). The Hallmarks of cancer. Cell Press.

[5] Tan Y., Wang Z., Xu M., Li B., Huang Z., Qin S., Nice E.C., Tang J., Huang C. (2023). Oral squamous cell carcinomas: State of the field and emerging directions. Int. J. Oral Sci..

[6] Pillay B., Wootten A.C., Crowe H., Corcoran N., Tran B., Bowden P., Crowe J., Costello A.J. (2016). The impact of multidisciplinary team meetings on patient assessment, management and outcomes in oncology settings: A systematic review of the literature. Cancer Treat. Rev..

[7] Zygogianni A.G., Kyrgias G., Karakitsos P., Psyrri A., Kouvaris J., Kelekis N., Kouloulias V. (2011). Oral squamous cell cancer: Early detection and the role of alcohol and smoking. Head Neck Oncol..

[8] Johnson D.E., Burtness B., Leemans C.R., Lui V.W.Y., Bauman J.E., Grandis J.R. (2020). Head and neck squamous cell carcinoma. Nat. Rev. Dis. Primers.

[9] Badwelan M., Muaddi H., Ahmed A., Lee K.T., Tran S.D. (2023). Oral Squamous Cell Carcinoma and Concomitant Primary Tumors, What Do We Know? A Review of the Literature. Curr. Oncol..

[10] Thomson P.J. (2018). Perspectives on oral squamous cell carcinoma prevention-proliferation, position, progression and prediction. J. Oral Pathol. Med..

[11] Nyalali A.M.K., Leonard A.U., Xu Y., Li H., Zhou J., Zhang X., Rugambwa T.K., Shi X., Li F. (2023). CD147: An integral and potential molecule to abrogate hallmarks of cancer. Front. Oncol..

[12] Barillari G., Melaiu O., Gargari M., Pomella S., Bei R., Campanella V. (2022). The Multiple Roles of CD147 in the Development and Progression of Oral Squamous Cell Carcinoma: An Overview. Int. J. Mol. Sci..

[13] de la Cruz Concepción B., Bartolo-García L.D., Tizapa-Méndez M.D., Martínez-Vélez M., Valerio-Diego J.J., Illades-Aguiar B., Salmerón-Bárcenas E.G., Ortiz-Ortiz J., Torres-Rojas F.I., Mendoza-Catalán M. (2022). EMMPRIN is an emerging protein capable of regulating cancer hallmarks. Eur. Rev. Med. Pharmacol. Sci..

[14] Warburg O. (1925). The Metabolism of Carcinoma Cells1. J. Cancer Res..

[15] Landras A., Reger de Moura C., Jouenne F., Lebbe C., Menashi S., Mourah S. (2019). CD147 Is a Promising Target of Tumor Progression and a Prognostic Biomarker. Cancers.

[16] Liu M., Tsang J.Y.S., Lee M., Ni Y.B., Chan S.K., Cheung S.Y., Hu J., Hu H., Tse G.M.K. (2018). CD147 expression is associated with poor overall survival in chemotherapy treated triple-negative breast cancer. J. Clin. Pathol..

[17] Huang Z., Wang L., Wang Y., Zhuo Y., Li H., Chen J., Chen W. (2013). Overexpression of CD147 contributes to the chemoresistance of head and neck squamous cell carcinoma cells. J. Oral Pathol. Med..

[18] Grass G.D., Dai L., Qin Z., Parsons C., Toole B.P. (2014). CD147: Regulator of hyaluronan signaling in invasiveness and chemoresistance. Adv. Cancer Res..

[19] Dai L., Guinea M.C., Slomiany M.G., Bratoeva M., Grass G.D., Tolliver L.B., Maria B.L., Toole B.P. (2013). CD147-Dependent Heterogeneity in Malignant and Chemoresistant Properties of Cancer Cells. Am. J. Pathol..

[20] Li L., Tang W., Wu X., Karnak D., Meng X., Thompson R., Hao X., Li Y., Qiao X.T., Lin J. (2013). HAb18G/CD147 Promotes pSTAT3-Mediated Pancreatic Cancer Development via CD44s. Clin. Cancer Res..

[21] Eghbalifard N., Nouri N., Rouzbahani S., Bakhshi M., Ghasemi Kahrizsangi N., Golafshan F., Abbasi F. (2025). Hypoxia signaling in cancer: HIF-1α stimulated by COVID-19 can lead to cancer progression and chemo-resistance in oral squamous cell carcinoma (OSCC). Discov. Oncol..

[22] Chen M., Liu Z., Zheng K., Hu C., Peng P. (2024). The potential mechanism of HIF-1α and CD147 in the development of triple-negative breast cancer. Medicine.

[23] Huang C., Sun Z., Sun Y., Chen X., Zhu X., Fan C., Liu B., Zhao Y., Zhang W. (2012). Association of increased ligand cyclophilin A and receptor CD147 with hypoxia, angiogenesis, metastasis and prognosis of tongue squamous cell carcinoma. Histopathology.

[24] Hanahan D., Weinberg R.A. (2011). Hallmarks of cancer: The next generation. Cell.

[25] Schürch C.M., Bhate S.S., Barlow G.L., Phillips D.J., Noti L., Zlobec I., Chu P., Black S., Demeter J., McIlwain D.R. (2020). Coordinated Cellular Neighborhoods Orchestrate Antitumoral Immunity at the Colorectal Cancer Invasive Front. Cell.

[26] Blatt S., Krüger M., Ziebart T., Sagheb K., Schiegnitz E., Goetze E., Al-Nawas B., Pabst A.M. (2017). Biomarkers in diagnosis and therapy of oral squamous cell carcinoma: A review of the literature. J. Cranio-Maxillofac. Surg..

[27] Muramatsu T. (2016). Basigin (CD147), a multifunctional transmembrane glycoprotein with various binding partners. J. Biochem..

[28] Xin X., Zeng X., Gu H., Li M., Tan H., Jin Z., Hua T., Shi R., Wang H. (2016). CD147/EMMPRIN overexpression and prognosis in cancer: A systematic review and meta-analysis. Sci. Rep..

[29] Belton R.J., Chen L., Mesquita F.S., Nowak R.A. (2008). Basigin-2 Is a Cell Surface Receptor for Soluble Basigin Ligand. J. Biol. Chem..

[30] Li L., Dong X., Peng F., Shen L. (2018). Integrin β1 regulates the invasion and radioresistance of laryngeal cancer cells by targeting CD147. Cancer Cell Int..

[31] Ju X., Liang S., Zhu J., Ke G., Wen H., Wu X. (2016). Extracellular matrix metalloproteinase inducer (CD147/BSG/EMMPRIN)-induced radioresistance in cervical cancer by regulating the percentage of the cells in the G2/m phase of the cell cycle and the repair of DNA Double-strand Breaks (DSBs). Am. J. Transl. Res..

[32] Kondo Y., Suzuki S., Takahara T., Ono S., Goto M., Miyabe S., Sugita Y., Ogawa T., Ito H., Satou A. (2021). Improving function of cytotoxic T-lymphocytes by transforming growth factor-β inhibitor in oral squamous cell carcinoma. Cancer Sci..

[33] Qiao B., Huang J., Mei Z., Lam A.K.-y., Zhao J., Ying L. (2020). Analysis of Immune Microenvironment by Multiplex Immunohistochemistry Staining in Different Oral Diseases and Oral Squamous Cell Carcinoma. Front. Oncol..

[34] Starska-Kowarska K. (2023). The Role of Different Immunocompetent Cell Populations in the Pathogenesis of Head and Neck Cancer—Regulatory Mechanisms of Pro- and Anti-Cancer Activity and Their Impact on Immunotherapy. Cancers.

[35] Vivier E., Raulet D.H., Moretta A., Caligiuri M.A., Zitvogel L., Lanier L.L., Yokoyama W.M., Ugolini S. (2011). Innate or Adaptive Immunity? The Example of Natural Killer Cells. Science.

[36] Cheng W., Lai P., Liu X., Wang Y., Du X. (2026). Lactate as a Metabolic Regulator in the Tumor Microenvironment: Linking Immunosuppression to Epigenetic Reprogramming. Curr. Pharm. Biotechnol..

[37] Liu Z., Zhang Z., Zhang Y., Zhou W., Zhang X., Peng C., Ji T., Zou X., Zhang Z., Ren Z. (2024). Spatial transcriptomics reveals that metabolic characteristics define the tumor immunosuppression microenvironment via iCAF transformation in oral squamous cell carcinoma. Int. J. Oral Sci..

[38] Lescaille G., Menashi S., Cavelier-Balloy B., Khayati F., Quemener C., Podgorniak M.P., Naïmi B., Calvo F., Lebbe C., Mourah S. (2012). EMMPRIN/CD147 up-regulates urokinase-type plasminogen activator: Implications in oral tumor progression. BMC Cancer.

[39] Cao Z., Xiang J., Li C. (2009). Expression of extracellular matrix metalloproteinase inducer and enhancement of the production of matrix metalloproteinase-1 in tongue squamous cell carcinoma. Int. J. Oral Maxillofac. Surg..

[40] Wang K., Chen X., Lin P., Wu J., Huang Q., Chen Z.-N., Tian J., Wang H., Tian Y., Shi M. (2024). CD147-K148me2-Driven Tumor Cell-Macrophage Crosstalk Provokes NSCLC Immunosuppression via the CCL5/CCR5 Axis. Adv. Sci..

[41] Gong J., Xie Y., Weng Z., Wu Y., Li L., Li B. (2026). Targeting Lactate and Lactylation in Cancer Metabolism and Immunotherapy. Adv. Sci..

[42] Deenadhayalan S.S., Edwin E.R., Elumalai K. (2024). Immunotherapy for Head and Neck Cancer: Mechanisms, Challenges, and Future Perspectives. J. Bio-X Res..

[43] Zhou J., Huang S., Wang L., Yuan X., Dong Q., Zhang D., Wang X. (2017). Clinical and prognostic significance of HIF-1α overexpression in oral squamous cell carcinoma: A meta-analysis. World J. Surg. Oncol..

[44] Li X.F., Yu X.Z., Dai D., Song X.Y., Xu W.G. (2016). The altered glucose metabolism in tumor and a tumor acidic microenvironment associated with extracellular matrix metalloproteinase inducer and monocarboxylate transporters. Oncotarget.

[45] Nagarathna P.J., Patil S.R., Veeraraghavan V.P., Daniel S., Aileni K.R., Karobari M.I. (2025). Oral cancer stem cells: A comprehensive review of key drivers of treatment resistance and tumor recurrence. Eur. J. Pharmacol..

[46] Jin L., Chun J., Pan C., Li D., Lin R., Alesi G.N., Wang X., Kang H.-B., Song L., Wang D. (2018). MAST1 Drives Cisplatin Resistance in Human Cancers by Rewiring cRaf-Independent MEK Activation. Cancer Cell.

[47] Li C.-X., Sun J.-L., Gong Z.-C., Lin Z.-Q., Liu H. (2016). Prognostic value of GLUT-1 expression in oral squamous cell carcinoma: A prisma-compliant meta-analysis. Medicine.

[48] Wang Y., Li Y., Jiang L., Ren X., Cheng B., Xia J. (2021). Prognostic value of glycolysis markers in head and neck squamous cell carcinoma: A meta-analysis. Aging.

[49] Zhu J., Wu Y.-N., Zhang W., Zhang X.-M., Ding X., Li H.-Q., Geng M., Xie Z.-Q., Wu H.-M. (2014). Monocarboxylate Transporter 4 Facilitates Cell Proliferation and Migration and Is Associated with Poor Prognosis in Oral Squamous Cell Carcinoma Patients. PLoS ONE.

[50] Katayama A., Bandoh N., Kishibe K., Takahara M., Ogino T., Nonaka S., Harabuchi Y. (2004). Expressions of Matrix Metalloproteinases in Early-Stage Oral Squamous Cell Carcinoma as Predictive Indicators for Tumor Metastases and Prognosis. Clin. Cancer Res..

[51] Magnussen S., Rikardsen O.G., Hadler-Olsen E., Uhlin-Hansen L., Steigen S.E., Svineng G. (2014). Urokinase Plasminogen Activator Receptor (uPAR) and Plasminogen Activator Inhibitor-1 (PAI-1) Are Potential Predictive Biomarkers in Early Stage Oral Squamous Cell Carcinomas (OSCC). PLoS ONE.

[52] Christensen A., Kiss K., Lelkaitis G., Juhl K., Persson M., Charabi B.W., Mortensen J., Forman J.L., Sørensen A.L., Jensen D.H. (2017). Urokinase-type plasminogen activator receptor (uPAR), tissue factor (TF) and epidermal growth factor receptor (EGFR): Tumor expression patterns and prognostic value in oral cancer. BMC Cancer.

[53] Huang W., Zhong L., Shi Y., Ma Q., Yang X., Zhang H., Zhang J., Wang L., Wang K., Li J. (2025). An Anti-CD147 Antibody−Drug Conjugate Mehozumab-DM1 is Efficacious Against Hepatocellular Carcinoma in Cynomolgus Monkey. Adv. Sci..

[54] Halford S., Veal G.J., Wedge S.R., Payne G.S., Bacon C.M., Sloan P., Dragoni I., Heinzmann K., Potter S., Salisbury B.M. (2023). A Phase I Dose-escalation Study of AZD3965, an Oral Monocarboxylate Transporter 1 Inhibitor, in Patients with Advanced Cancer. Clin. Cancer Res..

[55] Suzuki S., Ishikawa K. (2014). Combined inhibition of EMMPRIN and epidermal growth factor receptor prevents the growth and migration of head and neck squamous cell carcinoma cells. Int. J. Oncol..

[56] Li J., Ma C., Cao P., Guo W., Wang P., Yang Y., Ding B., Yin F., Li Z., Wang Y. (2025). A CD147-targeted small-molecule inhibitor potentiates gemcitabine efficacy by triggering ferroptosis in pancreatic ductal adenocarcinoma. Cell Rep. Med..

[57] Erber R., Spoerl S., Mamilos A., Krupar R., Hartmann A., Ruebner M., Taxis J., Wittenberg M., Reichert T.E., Spanier G. (2021). Impact of Spatially Heterogeneous Trop-2 Expression on Prognosis in Oral Squamous Cell Carcinoma. Int. J. Mol. Sci..

[58] Lydiatt W.M., Patel S.G., O’Sullivan B., Brandwein M.S., Ridge J.A., Migliacci J.C., Loomis A.M., Shah J.P. (2017). Head and Neck cancers-major changes in the American Joint Committee on cancer eighth edition cancer staging manual. CA Cancer J. Clin..

[59] Kononen J., Bubendorf L., Kallionimeni A., Bärlund M., Schraml P., Leighton S., Torhorst J., Mihatsch M.J., Sauter G., Kallionimeni O.-P. (1998). Tissue microarrays for high-throughput molecular profiling of tumor specimens. Nat. Med..

[60] Eichberger J., Spoerl S., Spanier G., Erber R., Taxis J., Schuderer J., Ludwig N., Fiedler M., Nieberle F., Ettl T. (2022). TIGIT Expression on Intratumoral Lymphocytes Correlates with Improved Prognosis in Oral Squamous Cell Carcinoma. Biomedicines.

